# General practitioners experience multi-level barriers to implementing recommended care for hip and knee osteoarthritis: a qualitative study

**DOI:** 10.1186/s12875-024-02658-0

**Published:** 2024-12-19

**Authors:** Alison J. Gibbs, Christian J. Barton, Nicholas F. Taylor, Joanne L. Kemp, Jason A. Wallis, Jo-Anne Manski-Nankervis, Allison M. Ezzat

**Affiliations:** 1 La Trobe Sports and Exercise Medicine Research Centre, School of Allied Health, Human Services and Sport, La Trobe University, Bundoora, Australia; 2 Department of Physiotherapy, Podiatry, Prosthetics and Orthotics, School of Allied Health, Human Services and Sport, La Trobe University, Bundoora, Australia; 3 Physiotherapy Department, Eastern Health, Box Hill Hospital, 8 Arnold Street, Box Hill, VIC 3128 Australia; 4 Allied Health Clinical Research Office, Eastern Health, Box Hill, Australia; 5 School of Public Health & Preventive Medicine, Monash University, Melbourne, Australia; 6 Primary Care and Family Medicine, Lee Kong Chian School of Medicine, Nanyang Technological University, Singapore, Singapore; 7 Department of General Practice and Primary Care, Melbourne Medical School, University of Melbourne, Melbourne, Australia; 8 Department of Physical Therapy, Faculty of Medicine, University of British Columbia, Vancouver, BC Canada

**Keywords:** Osteoarthritis management, General practitioner, Physiotherapy, Barriers, Qualitative

## Abstract

**Background:**

General practitioners (GPs) play a key role in managing osteoarthritis, including referring to appropriate management services. Physiotherapist-led osteoarthritis management programs and advanced practice triage services are effective, but GPs views on them are largely unknown. This study aimed to explore general practitioner perspectives on: (1) managing patients with hip and knee osteoarthritis, and (2) physiotherapy-led osteoarthritis care and referral pathways.

**Methods:**

Interview topic guides were developed based on the theoretical domains framework. Twenty-five semi-structured interviews with GPs were conducted. All data were coded independently by at least two researchers and analysed inductively using thematic analysis, with barrier themes mapped to the socioecological model.

**Results:**

Two interrelated themes were identified: (i) GPs had good general knowledge of recommended osteoarthritis care, but (ii) they faced multi-level challenges facilitating or directly providing evidence-based care. Nearly all GPs identified exercise as first-line care and surgery as a last resort. Most were aware imaging was not required to diagnose osteoarthritis, yet reported often referring for imaging. Many GPs expressed challenges facilitating patient engagement in physiotherapy due to patient, environmental/social and system level barriers. Key barriers included: perceived patient expectations and lack of motivation to attend physiotherapy, a lack of knowledge of available physiotherapy services, a lack of affordable physiotherapy services, and lengthy waiting times for public orthopaedic appointments. Having private health insurance was perceived as an enabler.

**Conclusions:**

Despite having good knowledge of guideline-recommended care, GPs in our study experienced multi-level barriers to implementing this care in practice. Public health information and strategies to address patient’s beliefs and lack of motivation to exercise may help reduce barriers to engaging in appropriate care. Urgent health system funding reforms are needed to allow GPs to appropriately manage patients with hip and knee osteoarthritis.

**Supplementary Information:**

The online version contains supplementary material available at 10.1186/s12875-024-02658-0.

## Background

General practitioners (GPs) have a crucial role managing and facilitating guideline-recommended care for people with hip and knee osteoarthritis, including arranging referral to other health professionals [1]. Higher-quality guidelines consistently recommend exercise, education and weight loss if required as first-line care for people with hip and knee osteoarthritis, with referral for arthroplasty only after adequate provision of first-line care [2]. However, GPs are more than twice as likely to refer to orthopaedic surgeons than refer for exercise [3]. Although guidelines recommend against opioids [2], GPs commonly prescribe opioids for osteoarthritis [3, 4]. Additionally, imaging is not recommended to diagnose osteoarthritis unless there is an atypical presentation [1, 5], but over 49% of people having an initial GP consultation are referred for imaging [3].

Referral to physiotherapy from a medical professional (GP or specialist physician) has been found to be an enabler for patients with hip and knee osteoarthritis to attend physiotherapy [6, 7], while a lack of referral is a key reason for people not accessing physiotherapy even when self-referral is available [6–8]. Numerous barriers hamper GP referral to first-line care [9, 10]. A lack of trust in the care provided by other health professionals, beliefs discordant with guidelines, short consultation times, inefficient referral processes, patient out of pocket costs, concerns regarding the safety and efficacy of exercise and a belief that surgery is the only option, have been identified as limiting GP adherence to guideline-recommend care [6, 10–13].

Physiotherapist-led osteoarthritis specific programs such as Good Life osteoArthritis: Denmark (GLA: D) [14] are reported to improve patients’ pain and function, reduce surgery desire [15], and may address some of the known barriers to guideline adherence. Advance practice physiotherapist-led models of care have been established in numerous countries, and have demonstrated high patient satisfaction, cost effectiveness, similar decision making to orthopaedic doctors, and positive impacts on waiting lists [16]. However, physiotherapist-led programs and advanced practice models of care are often underused.

General practitioner views on physiotherapist-led osteoarthritis services are largely unknown. One study explored barriers and enablers for referrals to, and participation in, a physiotherapist-led exercise and education program from the perspectives of patients, orthopaedic surgeons, rheumatologists and GPs [6]. It identified several common barriers (cost, misinformation about osteoarthritis, and lack of tailoring to individuals) and enablers (better program promotion, potential to avoid surgery) [6]. However, this study included patients and multiple medical professionals with only five GPs. Further, it sought their views on a specific program, not advanced practice physiotherapy or other aspects of osteoarthritis care [6]. A survey of Canadian primary care physicians found most agreed an advanced physiotherapy practice model for hip and knee osteoarthritis was useful, yet did not routinely refer to the program [17]. However, this study did not explore the barriers and enablers to referral [17]. There is also limited qualitative research considering GP thoughts on pharmacological and surgical management of osteoarthritis. Two recent studies explored medical professional’s views all on aspects of osteoarthritis management but were not specific to GPs [18, 19]. To the best of our knowledge, no qualitative research has been published exploring GP views on physiotherapist-led advanced practice osteoarthritis services. Understanding GP perceptions on all aspects of osteoarthritis management, including physiotherapist-led advanced practice and osteoarthritis management services, may assist in developing interventions to support GPs in facilitating guideline recommended care.

### Aims

We aimed to (i) explore GP beliefs and experiences of managing and referring patients with hip and knee osteoarthritis, and (ii) explore GP thoughts on physiotherapist-led osteoarthritis care and referral pathways, including advanced practice services.

## Participants and methods

### Study design

The philosophical orientation underpinning the qualitative approach was naturalistic enquiry, with key assumptions that (i) there are multiple realities that are subjective, (ii) the researcher and the research subjects interact to influence each other, and (iii) theory emerges from the data [20]. This qualitative study used an interpretive description approach, aiming for understanding of phenomena, not solely description [21]. The interpretive description approach was chosen as it is underpinned by the understanding that both psychosocial and biological phenomena influence human experience, with the researcher bringing their knowledge and experience to the process [22]. We used semi-structured interviews to explore GP experiences and beliefs regarding managing and referring patients with hip or knee osteoarthritis. To enhance the rigour, comprehensiveness and credibility, our study is reported consistent with Consolidated criteria for Reporting Qualitative research (COREQ) [23]. Ethics approval was received from La Trobe University (HEC21424). All participants provided written and verbal informed consent.

### Participants

General practitioner registrars and GPs working clinically in Victoria, Australia were recruited in three ways. First, GPs who were personal contacts of the research team were invited to participate. Second, GPs were recruited via advertisements in the newsletter of the VicREN primary care practice-based research network at Department of General Practice and Primary Care at The University of Melbourne. Any GPs who registered their interest were provided with further details of the study via email. General practitioners who expressed interest but did not return a signed consent form were contacted twice before considered not responding. Third, VicREN directly contacted GP practices. Sample size for recruitment was not specified beforehand. Rather, we interviewed until no further ideas or information were identified. Demographic details collected included age, gender, years of practice, setting (urban or regional) and frequency of seeing patients with hip or knee osteoarthritis.

### Data collection

Semi-structured interviews were conducted by a female registered physiotherapist working in an osteoarthritis hip and knee service, with previous qualitative research experience and training. The interviewer had no prior relationship with the GPs. The interviews were conducted either via zoom or telephone, and were audio recorded. Audio files were professionally transcribed verbatim, and transcripts checked for accuracy by the interviewer. Participants were offered a $AUD75 gift card as compensation for their time to participate.

Using a theoretical framework to guide data collection supports the use of common terminology, and systematic detection and classification of behaviour change factors [24]. A theory-based framework allows for comparisons and building of knowledge of determinants of behaviour, a requirement before considering developing behaviour change interventions [25]. The Theoretical Domains Framework (TDF) is a behaviour change framework that was developed by incorporating a number of models to facilitate identifying determinants of professional behaviour [26]. The TDF allows for considering a large range of factors that can influence behaviour including individual, social and environment/resources, and can be applied to develop behaviour change interventions using tools such as the Behaviour Change Wheel [27]. Initially, the TDF consisted of 12 key domains from 33 different theoretical models of behaviour [26] and was subsequently refined and validated to 14 key domains (Knowledge; Skills; Social/Professional Role and Identity; Beliefs about Capabilities; Optimism; Beliefs about Consequences; Reinforcement; Intentions; Goals; Memory, attention and Decision Processes; Environmental Context and Resources; Social Influences; Emotions; and Behavioural Regulation) [28].

In our current study, the TDF domains of behaviour change were discussed by the research team and informed the development of the interview guide to ensure it comprehensively considered diverse factors that influence behaviour. The TDF has been used in previous research to identify health professionals behaviour across a variety of settings [29], including knee osteoarthritis research [6, 19, 30]. The interview guide (see Additional file 1) was reviewed by one consumer with lived experience of osteoarthritis. The interview guide had two sections, covering (i) GPs views on osteoarthritis management, and (ii) learning preferences and views on a planned intervention to increase referrals to physiotherapy services (reported separately). The interviews evolved based on GP responses and discussion within the research team, with earlier interviews focusing mostly on GP thoughts on osteoarthritis management and barriers and enablers to first-line care. Later interviews focused more on specific osteoarthritis programs and referral pathways. When it was determined by discussion within the research team that no further ideas were being identified (commonly termed data saturation), an additional two interviews were conducted to confirm this, with no new themes generated.

### Data analysis

All transcripts were read through iteratively by Researcher A1 allowing for familiarization, while a sample of GP interview transcripts were reviewed iteratively by researchers A2 and A7 during the data collection process. Member checking was not performed due to interviewee time constraints. The interviews were discussed monthly with the research team allowing for evolution of the topic guide. Inductive thematic analysis was chosen as it allows for the exploration of new data, and the identification of common themes across different individual perspectives [31]. The six steps of thematic analysis were adhered to with initial familiarisation with the interview transcripts, followed by generating codes. One researcher (A1) reviewed and coded all transcripts, based on an inductive thematic analysis approach [32], supported by NVIVO software. Researchers A2 and A7 each reviewed and coded a random sample of 50% of the interviews, with three of the research team (A3, A4, and A5) also each reviewing a random sample of four interviews. Research team members A1, A2, A3, A4 A5 and A7 met six times to discuss the data and coding as interviews were occurring and search for early themes. The themes were then reviewed, defined, then named. Finally, all team members read and provided feedback on the report of the themes. During these discussions, the socioecological model [33] was inductively identified as an appropriate model on which to deductively map the barrier themes. The socioecological model considers the interrelationship between behaviour and environmental conditions (including physical, social and cultural dimensions), emphasizing the interaction of situational and personal factors, including environmental resources [34]. The socioecological model for health aims to explain human behaviour at multiple levels: intrapersonal, interpersonal, institutional, community, and public policy [33]. The barrier themes which were identified reflected these levels of influences on behaviour. Six researchers were experienced physiotherapists (range 18–39 years’ experience), one was a GP researcher. One (A1) was a PhD candidate who had attended a 2-day qualitative training course and undertaken previous qualitative research, and the remaining were all experienced researchers (range 4–23 years post PhD). Reflexivity was considered with A1 cognisant of her professional background and role as an advanced practice physiotherapist and potential for pre-conceived views to influence data analysis. To provide a balanced perspective, multiple members of the research team were involved in the data analysis and coded interviews independently. Further, supportive quotes to justify coding and themes were retained in NVivo. Additionally, having a GP as part of the research team and reviewing the themes further enhanced reflexivity.

## Results

### Participant characteristics

Twenty-eight GPs were contacted directly by the research team and invited to participate, and 422 practices were contacted by the primary based research network (Table 1). Twenty-six GPs consented and were interviewed. One declined interview audio recording, resulting in 25 included interview transcripts. Participants’ mean age was 47 years (SD 11). Most GPs worked in metropolitan practices, had greater than 5 years’ experience, and saw patients with osteoarthritis greater than 5 times a week (Table 2). Interviews lasted an average of 29 min (range: 15–52 min).

**Table 1:** Recruitment of General practitioners

	Contacted	Responded	Declined	Consented	Attended interview
Contacts within research team	17^#^	7^#^	1^#^	6	6
Responded to EOI advertisement	11^#^	5^#^	1^#^	2	2
VICRen	422*	258*	143*	22	18

Contacted = number of GPs/practices contacted; Responded = number of GPs/practices who responded to request to participate; Declined = Number of GPs/practices who declined to participate; Consented = number of GPs who returned signed consent forms; Attended interview = number of GPs who attended an interview; # = individual GP; * = GP practice; EOI = expression of interest from newsletter advertisement; VICRen = Victorian Primary Care Practice-Based Research Network

**Table 2:** Demographics of GPs

GP ID no	Gender	Age	Years in practice	No OA patients a week	Metro/regional
1	W	30–39	< 5	2–5	METRO
2	W	40–49	11–20	> 15	METRO
3	W	30–39	< 5	1	METRO
4	M	40–49	5–10	6–15	METRO
5	W	50–59	> 20	6–15	METRO
6	M	50–59	> 20	6–15	METRO
7	W	40–49	< 5	2–5	METRO
8	M	40–49	> 20	6–15	METRO
9	W	30–39	5–10	< 1	METRO
10	W	30–39	< 5	2–5	METRO
11	M	50–59	11–20	> 15	METRO
12	W	40–49	11–20	6–15	METRO
13	M	50–59	11–20	2–5	METRO
14	W	*	> 20	2–5	METRO
15	M	30–39	5–10	1	METRO
16	W	60–69	> 20	6–15	REGIONAL
17	M	60–69	> 20	6–15	METRO
18	M	60–69	> 20	> 15	METRO
19	M	40–49	5–10	2–5	METRO
20	M	50–59	11–20	6–15	METRO
21	M	40–49	11–20	2–5	METRO
22	M	60–69	> 20	> 15	REGIONAL
23	W	30–39	< 5 (gp REG)	< 1	METRO
24*	M	-	-	-	METRO
25	M	30–39	< 5 (gp REG)	6–15	REGIONAL

W = Woman, M = Man, Age = years, Metro = Metropolitan Melbourne, Regional = Regional Victoria

*=failed to provide demographic data

### Themes

Two interrelated themes were identified: *(i) GPs mostly have good general knowledge of recommended osteoarthritis care* and *(ii) GPs face multi-level challenges facilitating or directly providing evidence-based care.*

#### GPs mostly have good general knowledge of recommended osteoarthritis care

Most GPs reported beliefs aligning to current guideline recommendations, although some reported lacking certainty in the mechanisms of effectiveness, and implementation of, first-line care (see Supplementary Table 1, Additional file 2 for further quotes supporting theme 1).

GPs viewed exercise and weight loss as important first steps for managing hip and knee osteoarthritis, with most indicating they consider referring patients to physiotherapy for exercise prior to referring for orthopaedic opinion.

*Weight management, diet, and exercise are the mainstay of management, of course… The benefits are unchallenged, we know how effective it is. (GP24)*

Although GPs rated exercise highly, several expressed uncertainty regarding the benefits and type of exercise, and how to prescribe exercises.

*I personally don’t know if you do vigorous jumping and hopping exercise what will happen. (GP13)*

*Maybe I’ve not had the confidence [to prescribe exercises] ‘cause I can’t teach the exercise… if I don’t feel like I have the confidence to teach them properly, then I won’t. (GP5)*

While GPs agreed weight management was important, there were two distinct approaches. Several GPs raised concerns regarding the challenges of weight management, including difficulty accessing both allied health and weight loss medication. Some GPs highlighted concerns regarding the emotional impact of weight and wariness of weight stigmatisation, and described approaching weight management holistically by encouraging healthier lifestyles. Alternatively, a few GPs who viewed weight loss as necessary to reduce joint load, reported less concerns discussing weight loss.

*Approach [it] in a different way as opposed to just saying, “Look, we know that if you lost some weight, it probably would help,”… I talk to people less about weight management, and I talk more about healthy behaviours. (GP1)*

*Less load on the joints. It probably is something I’d mention, straight off, that keeping your weight as low as possible to take some weight off your joints. (GP5)*

Although most GPs stated imaging was not required to diagnose osteoarthritis, many reported they still commonly referred for imaging. Mostly this appeared related to wanting confirmation of the diagnosis and meeting patient expectation, with several GPs commenting patients wanted imaging.

*Well, mostly [do arrange x-ray]… Often you can probably, sort of, diagnose it even prior to the x-ray. The x-ray is just for confirmation. (GP11)*

*I just do an x-ray ‘cause everyone expects to have one done… so yeah, I’d usually do an x-ray. (GP23)*

Most GPs prescribed paracetamol as a primary pharmacology option due to its perceived safety profile. Several expressed awareness of a lack of efficacy of paracetamol, but they preferred its use due fewer side effects than alternatives. Non-steroidal anti-inflammatories were viewed as more effective in managing pain, but higher risk. Intraarticular injections were considered an adjunct. Management of severe pain was reported as challenging, with a perceived lack of effective options. Interviewees reported mostly avoiding opioid prescriptions.

*Medication role is really mainly with pain control and it’s certainly a very imperfect management strategy (GP22)*.

Surgery was generally considered a last resort with mixed views on its overall effectiveness.

*Well, it’s end-stage, really… I get a lot of people with knee surgery get improvement on level of pain and function but, certainly there are a few more people who aren’t helped as much as they might have expected to be. (GP16)*

#### GPs face challenges facilitating or directly providing evidence-based care

**Fig. 1:**
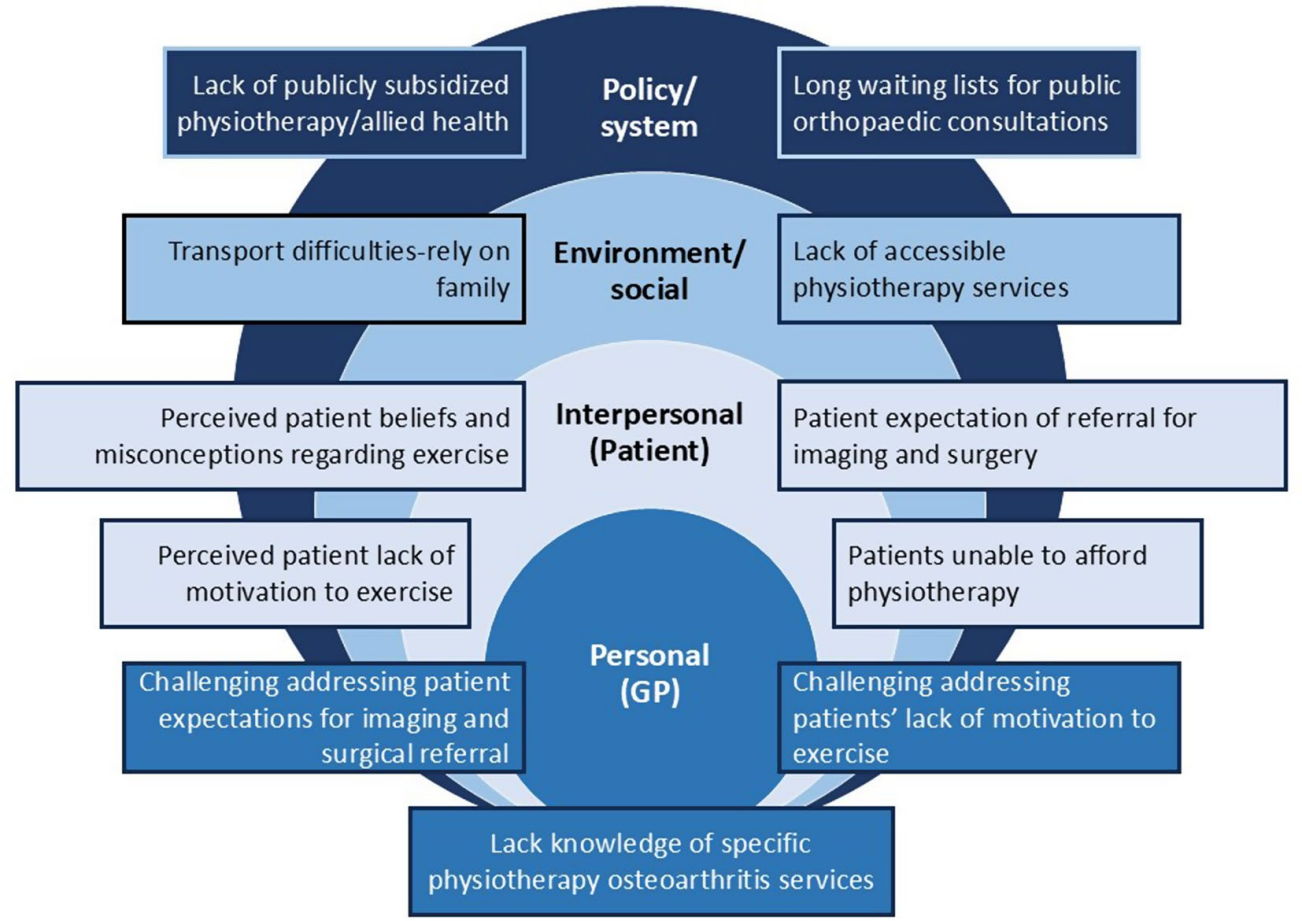
Barriers mapped to the socioecological model

Despite most having knowledge of guideline recommended care, GPs reported several challenges implementing this care in practice. Barriers were identified at intrapersonal (GP), interpersonal (patient), institutional and community (grouped together as environment/social) and public policy (policy/system) levels, and illustrated in Fig. 1. See Supplementary Table 2, Additional File 2 for further quotes supporting theme 2.

##### GP level

Many GPs reported challenges with how to encourage patients to participate in recommended care. GPs discussed a lack of time in consultations, and difficulty in addressing motivation in patients who were disinclined to exercise. Some GPs expressed uncertainty regarding the mechanisms for how exercise can be beneficial for people with osteoarthritis and how to communicate this to patients who were reluctant to exercise.

*People don’t quite understand the value of physio and exercise… They [patients] just want to passively have someone fix them. And so challenging that mindset I find difficult. (GP23)*

*A lot of patients want surgery and physio doesn’t cut it so, occasionally, I’ve even had to refer patients to surgeons so that the surgeon can tell them to go to the physio, because it seems to come better going from them (GP12)*

*I would love to understand what benefits patients can get through exercises, so I can convince them, because sometimes, they question, “Ah, would it do something or do anything that it would be beneficial?” (GP7)*

Most GPs had limited awareness of referral pathways and physiotherapist-led osteoarthritis-specific programs such as GLA: D^®^ or advanced practice physiotherapy osteoarthritis services. Having knowledge of outcomes of programs such as GLA: D^®^, including providing GPs with patient handouts, was suggested as a potential enabler to increasing patient willingness to attend physiotherapy.

*Patients do like visual information. So, if there was something you could show them with some figures or charts or something like that, that might be useful, what percentage of people benefits, what worked. (GP6)*

*Maybe some material we can give them. And the statistics that we can say like this many people have avoided joint replacements by doing this physio program (GP12)*

*I think patients as well, if you can say to them, “Oh, there’s this specific program designed for osteoarthritis”, that’s a lot more useful than saying, “Oh, go see a physio.” (GP3)*

When the advanced practice physiotherapy model of care was explained by the interviewer, GPs were mostly in favour. Multiple potential benefits were mentioned by GPs, including assisting in management, providing an alternative to orthopaedic surgeon referrals, reducing demand on surgeons, and facilitating access to surgeons when required. A community-based advanced practice model was thought to potentially improve patient accessibility, and positively influence patient expectations.

*If they could determine “Okay, actually, there’s better benefit from nonsurgical intervention, you know what, you don’t need to stay on this wait list for however many years, let’s get you started on whatever” or “Oh no, you really do need to be on the waitlist. Okay, let’s leave you on there.” I think that would be useful for sure, that would be great. (GP3)*

*The minute you mention going to the hospital, the people’s perception about hospitals is feeling that they are already end stage or debilitated. I would say someone in between GP as a primary care and the hospital system who would facilitate and classify and treat accordingly or promptly refer if need be, is a good approach. (GP4)*

However, a few GPs had concerns that an advanced practice physiotherapy service may delay care and create an additional hurdle for accessing orthopaedics.

*I don’t see the point of getting a second person to say yes or no. It’s almost like doing it twice. (GP24)*

##### Patient level

Patient misconceptions such as surgery being the only option and a lack of motivation to exercise and attend physiotherapy or dietetics were identified by most GPs as a major barrier to referring patients for non-operative management. Most patients were perceived to be uncertain of the benefits of exercise and expecting surgery. Willingness to engage with, and understanding the potential benefits of, physiotherapy and exercise were perceived as enablers to referral.

*The patient expects if they come in and they’ve got hip or knee pain, that they’re going to be referred to a surgeon and most of them are fully expecting that they’re going to get a joint replacement. (GP14)*

*People find it hard to believe that physiotherapy or muscle strengthening could actually help (GP14)*

*Patient’s motivations is a big factor, especially with things like seeing the physio, doing the exercises that the physio gives you… So, I think that’s the most challenging thing is, you have to have motivated patients, and it’s hard (GP3)*

Referral decision making was often guided by patient preference, with imaging and orthopaedic referrals reported to be made based on patient requests; as well as patients requesting not to be referred to physiotherapy or dietetics.

*If it’s some mild or moderate, that’s probably not much benefit of referring to a surgeon but it’s really patient-dependent; if they feel like they want an opinion, I’ll refer them if that’s the patient’s expectation (GP25)*

##### Environmental/social level

Accessibility of local physiotherapy services due to environmental factors, such as location and transport, was raised as a barrier by several GPs, especially those based in regional areas. Social factors, including transport difficulties when relying on family and timing of services, were mentioned by some GPs.

*Sometimes it’s not accessible enough for them and they would rather go to a physiotherapist or somebody who is easily accessible because either they don’t hold a licence, or they are dependent on other people to take them to the services. (GP2)*

##### Policy/system level

Almost all GPs raised cost as a barrier to patients accessing physiotherapy. Within publicly subsidised allied health options, two different frustrations were apparent. Care was either available quickly but for a limited number of sessions (≤ 5); or there were lengthy waiting times to access funding mechanisms to provide an adequate number of sessions. Despite the limitations and frustrations expressed, publicly subsidised allied health sessions were valued as helping with affordability. Patients being able to afford private physiotherapy was viewed as an enabler.

*Good physiotherapy costs money, and not all of my patients can afford that. (GP1)*

*It’s not as accessible if it’s not private (GP6)*

*Community health is really hard to get in with, so I use community health when we have to and we sort of just have to wait for the service to be available. (GP12)*

Lengthy public orthopaedic surgical waiting lists were a further source of dissatisfaction, with GPs expressing frustration when patients are unable to access the required care. Lengthy waiting times influenced referral decisions for some GPs who referred earlier or did multiple referrals to public orthopaedic services, and most preferred to refer privately if patients could afford the out-of-pocket consultation expenses, or patients had private health insurance. Private health insurance was perceived as an enabler for accessing orthopaedic surgical opinion.

*Oh, my goodness, long waiting… you can see that patients are desperate about the access to specialists and how long they wait. (GP4)*

*Sometimes I use another pathway. I ask some surgeons, “Please, see the patient in your room. Just charge whatever you charge for the consultation… And could you please put them under your public list for them?” We’re trying every possible way in order to help these people. So, the decision-makers have to do something. (GP13)*

*People who have got private insurance, they don’t have an issue, but people who have got public insurance, the only thing to be careful about is that you start it early. I refer to a couple of hospitals, that’s the other thing. So wherever the patient gets into first. (GP21)*

## Discussion

This study explored GP beliefs and experiences of managing and referring patients with hip and knee osteoarthritis, including their thoughts on physiotherapist-led osteoarthritis advanced practice and management services and referral pathways. Consistent with recent evaluations of GP knowledge [18, 35], GPs in this study mostly reported knowledge in line with higher-quality guideline recommendations [2]. Nearly all GPs believed exercise was important in the management of hip and knee osteoarthritis, with most being aware that imaging was not required to diagnose osteoarthritis, and believing surgery was a last resort. Yet, system-related challenges to arranging non-operative care, including a lack of publicly subsidized allied health, combined with multi-level challenges addressing patient expectations and motivation, rather than a lack of knowledge, appeared to hinder guideline adherence.

General practitioners expressed challenges in persuading patients to trial guideline-recommended care, with many interrelated factors across the socioecological model potentially contributing to this challenge. Although many GPs reported making referrals to exercise when their patients were open to it, they reported common challenges in facilitating patient engagement. Lack of patient motivation and patient beliefs contrary to evidence were perceived by GPs as key barriers to engagement with guideline recommended care. GPs expressed that patient beliefs often focused on surgery as inevitable and the only option, with exercise unlikely to help. These beliefs are consistent with an impairment discourse [36], which centers on osteoarthritis as a mechanical “wear and tear” problem requiring “fixing” [37], and may contribute to a subsequent lack of motivation to participate in exercise [13]. Frequent imaging reported by GPs in our study and previous qualitative [10] and quantitative research [38] may also contribute to an impairment discourse [39], particularly if terminology such as “bone on bone” is used in radiology reports and in discussion with patients. A multipronged approach from health professionals, media, social media, health institutions and public policy to change the messaging around osteoarthritis to a more participatory discourse is required. Changing the terminology used [40], and promoting messages such as surgery is not inevitable, and being active is safe despite changes on imaging [41], may better align patient beliefs with guideline recommended care.

Consistent with previous research [42, 43], patient preference influenced referral decisions of GPs related to imaging, orthopaedics, exercise, and weight management. Expectations of treatment effectiveness influences patient treatment choices for osteoarthritis [44], yet most patients believe they are not sufficiently educated on treatment options [45]. Tools such as patient decision aids and option grids may assist GPs in educating patients and informing their treatment choices [46, 47]. Interventions incorporating behaviour change strategies such as motivational interviewing [48] and option grids [47] may support GPs to enhance patient engagement with guideline recommended care, and warrant future evaluation.

Frustration with system-related barriers such as costs and public waiting times was evident and further impeded GPs adhering to guideline-recommended care. A lack of affordable and accessible options for non-operative management was commonly reported, with similar views expressed previously by patients and multiple differing health professionals from numerous countries [6, 10, 13, 49, 50]. Adequate funding of physiotherapy-led programs is needed and may assist in reducing the escalating burden of hip and knee osteoarthritis to the health system [51]. The lack of publicly funded physiotherapy options combined with concerns regarding lengthy public orthopedic waiting lists influenced referral decision making. GPs described referring to orthopaedics earlier than indicated clinically, with the perception patients will require surgery eventually. However, osteoarthritis is not an inevitable decline to the point of requiring arthroplasty [52], meaning such referral behavior may unnecessarily add to growing waiting lists and further compound the prevailing impairment discourse. Up to two-thirds of patients referred by GPs to orthopedics do not get waitlisted for surgery [53]. Advanced practice physiotherapists can act as triaging clinicians, offering similar decision making to surgeons [54]. Despite many of our GPs lacking familiarity with advanced practice physiotherapy clinics, most were positive towards the concept of this service. Increasing GP referral of patients to physiotherapist-led services, including advanced practice physiotherapy, may help to reduce current long wait lists for public orthopedic services [16, 55].

Our GPs demonstrated limited awareness of both physiotherapist-led osteoarthritis management programs and advanced practice services. Structured physiotherapist-led non-operative programs appear to be cost effective [56, 57], improve patient outcomes [14, 58], and may delay or prevent the need for surgery [15, 59]. Despite the benefits of physiotherapist-led osteoarthritis programs, including advanced practice services, they remain underused. Patient treatment decision making choices may be influenced by their GP [44, 60], with doctor referral an enabler to patients attending physiotherapy [6]. Increasing GP and community awareness of physiotherapist-led osteoarthritis management programs and their outcomes may assist in increasing patient motivation to attend physiotherapy as identified by some of our GPs.

Our study has a number of strengths, as well as some limitations to consider. We conducted a large number of GP interviews, capturing the perspectives of GPs with a range of years of experience, age, frequency of OA management, and a mix of metropolitan and regional GPs. The use of validated theoretical frameworks and models to inform the interview guide during data collection and map the themes during analysis strengthened our methods and results. It helps ensure factors influencing behaviour are identified which can subsequently be used to develop a behaviour change intervention [25]. To the best of our knowledge this is the first study to specifically investigate GP views on all aspects of osteoarthritis management, including advanced practice physiotherapy services. Most participants in this study worked in metropolitan areas and none were based in remote areas. This largely reflects the demographics of GPs in Victoria, with 93% of GPs either metropolitan or rural [61]. The views of remote location GPs and those in other health services or locations may differ from the GPs in our sample. A large number of GPs and GP practices were contacted to participate in this study but declined. Given the low response rate, it is possible GPs who elected to participate have a greater interest and understanding in osteoarthritis and differing beliefs from GPs who did not participate. Additionally, responder bias may have been further influenced by participating GPs being aware the interviewer was a physiotherapist. Transcripts were not member checked, and the majority of the research team were physiotherapists, however a GP was also involved in developing the themes.

## Conclusion

The GPs in our study reported knowledge of current guideline-recommended care but experienced multi-level challenges to implement this care in practice. The biggest challenges for GPs appear to be managing patient expectations and motivations to allow for guideline-based care and being able to identify accessible physiotherapy-led services for their patients. Strategies to increase patient engagement in appropriate care such as further public health information initiatives, increased awareness of physiotherapist-led osteoarthritis programs among GPs and the community, and greater support to address patient’s lack of motivation to attend physiotherapy are needed. Urgent health system funding reforms providing timely publicly-funded non-operative management programs are needed to improve access to first-line care.

## Electronic supplementary material

Below is the link to the electronic supplementary material.

Supplementary Material 1: Interview topic guide

Supplementary Material 2: Supplementary material additional quotes. Supplementary Table 1: Additional Quotes Theme 1 GPs have good general knowledge of recommended care; and Supplementary Table 2: Additional Quotes Theme 2 General practitioners face challenges facilitating or directly providing evidence-based care

Supplementary Material 3

## Data Availability

The authors declare that the data supporting the findings of this study are available within the paper, and its supplementary information files. Full transcripts of the interviews are not published as contain some identifying information, however will be available on reasonable request. Data is stored in a controlled access research drive at La Trobe University.

## References

[1] The Royal Australian College of General Practitioners. Guideline for the management of knee and hip OA 2nd edition East Melbourne: RACGP. 2018. https://www.racgp.org.au/download/Documents/Guidelines/Musculoskeletal/guideline-for-the-management-of-knee-and-hip-oa-2nd-edition.pdf

[2] Gibbs AJ, Gray B, Wallis JA, Taylor NF, Kemp JL, Hunter DJ, et al. Recommendations for the management of hip and knee osteoarthritis: a systematic review of clinical practice guidelines. Osteoarthritis Cartilage. 2023;31(10):1280–92. 10.1016/j.joca.2023.05.015. 37394226 10.1016/j.joca.2023.05.015

[3] Bennell KL, Bayram C, Harrison C, Brand C, Buchbinder R, Haas R, et al. Trends in management of hip and knee osteoarthritis in general practice in Australia over an 11-year window: a nationwide cross-sectional survey. Lancet Reg Health West Pac. 2021;12:100187. 10.1016/j.lanwpc.2021.100187. 34527976 10.1016/j.lanwpc.2021.100187 PMC8356093

[4] Khoja SS, Almeida GJ, Freburger JK. Research. Recommendation rates for physical therapy, Lifestyle Counseling, and Pain medications for managing knee osteoarthritis in Ambulatory Care settings: a cross-sectional analysis of the National Ambulatory Care Survey (2007–2015). Arthritis Care Res (Hoboken). 2020;72(2):184–92. 31595710 10.1002/acr.24064

[5] National Institute for Health and Care Excellence. Osteoarthritis in over 16’s: diagnosis and management. 2022. Contract No.: NG226. 36745715

[6] Wallis JA, Ackerman IN, Brusco NK, Kemp JL, Sherwood J, Young K, et al. Barriers and enablers to uptake of a contemporary guideline-based management program for hip and knee osteoarthritis: a qualitative study. Osteoarthr Cartil Open. 2020;2(4):100095. 10.1016/j.ocarto.2020.100095. 36474878 10.1016/j.ocarto.2020.100095 PMC9718255

[7] Christiansen MB, Dix C, Master H, Jakiela JT, Habermann B, Silbernagel KG, et al. I’ve been to physical therapy before, but not for the knees. A qualitative study exploring barriers and facilitators to physical therapy utilization for knee osteoarthritis. Musculoskelet Care. 2020;18(4):477–86. 10.1002/msc.1491. 10.1002/msc.1491 PMC7749817 32588487

[8] Bopf D, McAuliffe M, Shillington M, Drynan D, Bucknell E. Knee osteoarthritis: use of investigations and non-operative management in the Australian primary care setting. Australasian Med J (Online). 2010;1(3):194–7.

[9] Okwera A, May S. Views of general practitioners toward physiotherapy management of osteoarthritis—a qualitative study. Physiother Theory Pract. 2019;35(10):940–6. 10.1080/09593985.2018.1459987. 29658794 10.1080/09593985.2018.1459987

[10] Egerton T, Nelligan RK, Setchell J, Atkins L, Bennell KL. General practitioners’ views on managing knee osteoarthritis: a thematic analysis of factors influencing clinical practice guideline implementation in primary care. BMC Rheumatol. 2018;2(30). 10.1186/s41927-018-0037-4. 10.1186/s41927-018-0037-4 PMC6390779 30886980

[11] Egerton T, Diamond LE, Buchbinder R, Bennell KL, Slade SC. A systematic review and evidence synthesis of qualitative studies to identify primary care clinicians’ barriers and enablers to the management of osteoarthritis. Osteoarthr Cartil. 2017;25(5):625–38. 10.1016/j.joca.2016.12.002. 10.1016/j.joca.2016.12.002 27939622

[12] Christiansen MB, White DK, Christian J, Waugh E, Gakhal N, King L, et al. It … doesn’t always make it [to] the top of the list. Can Fam Physician. 2020;66(1):e14.

[13] Miller KA, Osman F, Baier Manwell L. Patient and physician perceptions of knee and hip osteoarthritis care: a qualitative study. Int J Clin Pract. 2020;74(12):e13627. 10.1111/ijcp.13627. 32734667 10.1111/ijcp.13627

[14] Skou ST, Roos EM. Good life with osteoArthritis in Denmark (GLA:D™): evidence-based education and supervised neuromuscular exercise delivered by certified physiotherapists nationwide. BMC Musculoskelet Disord. 2017;18. 10.1186/s12891-017-1439-y. 10.1186/s12891-017-1439-y PMC5297181 28173795

[15] Barton CJ, Kemp JL, Roos EM, Skou ST, Dundules K, Pazzinatto MF, et al. Program evaluation of GLA:D^®^ Australia: physiotherapist training outcomes and effectiveness of implementation for people with knee osteoarthritis. Osteoarthr Cartil Open. 2021;3(3):100175. 10.1016/j.ocarto.2021.100175. 36474815 10.1016/j.ocarto.2021.100175 PMC9718148

[16] Samsson KS, Grimmer K, Larsson MEH, Morris J, Bernhardsson S. Effects on health and process outcomes of physiotherapist-led orthopaedic triage for patients with musculoskeletal disorders: a systematic review of comparative studies. BMC Musculoskelet Disord. 2020;21(1):673. 10.1186/s12891-020-03673-9. 33038935 10.1186/s12891-020-03673-9 PMC7548042

[17] de Sa D, Petruccelli D, Patton SJ, Winemaker M, de Beer J. From surgical gatekeepers to patient navigators: examining perceptions and practices of hip and knee osteoarthritis management among primary healthcare physicians. Curr Orthop Pract. 2016;27(1):46–55. 10.1097/BCO.0000000000000290.

[18] Sutton L, Jose K, Betzold A, Hansen E, Laslett L, Makin J, et al. Understanding the management of osteoarthritis: a qualitative study of GPs and orthopaedic surgeons in Tasmania, Australia. Osteoarthr Cartil Open. 2021;3(4):100218. 10.1016/j.ocarto.2021.100218. 36474752 10.1016/j.ocarto.2021.100218 PMC9718107

[19] Wallis JA, Barton CJ, Brusco NK, Kemp JL, Sherwood J, Young K, et al. Exploring views of orthopaedic surgeons, rheumatologists and general practitioners about osteoarthritis management. Musculoskelet Care. 2021;19(4):524–32. 10.1002/msc.1549. 10.1002/msc.1549 PMC9292668 33710743

[20] Guba EG. ERIC/ECTJ Annual Review Paper: Criteria for assessing the trustworthiness of naturalistic inquiries. Educational Communication Technol. 1981;29(2):75–91.

[21] Thorne S, Kirkham SR, MacDonald-Emes J. Interpretive description: a noncategorical qualitative alternative for developing nursing knowledge. Res Nurs Health. 1997;20(2):169–77. https://doi.org/10.1002/(sici)1098-240x(199704)20:2<169::aid-nur9>3.0.co;2-i. 9100747 10.1002/(sici)1098-240x(199704)20:2<169::aid-nur9>3.0.co;2-i

[22] Thompson Burdine J, Thorne S, Sandhu G. Interpretive description: a flexible qualitative methodology for medical education research. Med Educ. 2021;55(3):336–43. 10.1111/medu.14380. 32967042 10.1111/medu.14380

[23] Tong A, Sainsbury P, Craig J. Consolidated criteria for reporting qualitative research (COREQ): a 32-item checklist for interviews and focus groups. Int J Qual Health Care. 2007;19(6):349–57. 17872937 10.1093/intqhc/mzm042

[24] Noar SM, Zimmerman RS. Health Behavior Theory and cumulative knowledge regarding health behaviors: are we moving in the right direction? Health Educ Res. 2005;20(3):275–90. 10.1093/her/cyg113. 15632099 10.1093/her/cyg113

[25] Cathain A, Croot L, Duncan E, Rousseau N, Sworn K, Turner KM, et al. Guidance on how to develop complex interventions to improve health and healthcare. BMJ Open. 2019;9(8):e029954. 10.1136/bmjopen-2019-029954. 10.1136/bmjopen-2019-029954 PMC6701588 31420394

[26] Michie S, Johnston M, Abraham C, Lawton R, Parker D, Walker A. Making psychological theory useful for implementing evidence based practice: a consensus approach. BMJ Qual Saf. 2005;14(1):26–33. 10.1136/qshc.2004.011155. %J Quality and Safety in Health Care. 10.1136/qshc.2004.011155 PMC1743963 15692000

[27] Michie S, van Stralen MM, West R. The behaviour change wheel: a new method for characterising and designing behaviour change interventions. Implement Sci. 2011;6(1):42. 10.1186/1748-5908-6-42. 21513547 10.1186/1748-5908-6-42 PMC3096582

[28] Cane J, O’Connor D, Michie S. Validation of the theoretical domains framework for use in behaviour change and implementation research. Implement Sci. 2012;7(1):37. 10.1186/1748-5908-7-37. 22530986 10.1186/1748-5908-7-37 PMC3483008

[29] Phillips CJ, Marshall AP, Chaves NJ, Jankelowitz SK, Lin IB, Loy CT, et al. Experiences of using the theoretical domains Framework across diverse clinical environments: a qualitative study. J Multidisciplinary Healthc. 2015;8(null):139–46. 10.2147/JMDH.S78458. 10.2147/JMDH.S78458 PMC4370908 25834455

[30] Bahns C, Happe L, Kopkow C. Barriers and facilitators to the use of clinical practice guidelines in osteoarthritis care: a qualitative study among German physiotherapists. BMJ Open. 2024;14(10):e085349. 10.1136/bmjopen-2024-085349. 39424395 10.1136/bmjopen-2024-085349 PMC11492947

[31] Vaismoradi M, Turunen H, Bondas T. Content analysis and thematic analysis: implications for conducting a qualitative descriptive study. Nurs Health Sci. 2013;15(3):398–405. 23480423 10.1111/nhs.12048

[32] Braun V, Clarke V. Using thematic analysis in psychology. Qual Res Psychol. 2006;3(2):77–101. 10.1191/1478088706qp063oa.

[33] McLeroy KR, Bibeau D, Steckler A, Glanz K. An ecological perspective on health promotion programs. Health Educ Q. 1988;15(4):351–77. 10.1177/109019818801500401. 3068205 10.1177/109019818801500401

[34] Stokols D. Translating social ecological theory into guidelines for community health promotion. Am J Health Promot. 1996;10(4):282–98. 10.4278/0890-1171-10.4.282. 10159709 10.4278/0890-1171-10.4.282

[35] O’Leary H, Robinson K, Glynn L, Lenehan B, McCreesh K. You’re stuck in the middle here: a qualitative study of GPs’ experiences of managing knee pain attributed to a degenerative meniscal tear. BMC Prim Care. 2023;24(1):127. 10.1186/s12875-023-02075-9. 37344762 10.1186/s12875-023-02075-9 PMC10286499

[36] Bunzli S, Taylor N, O’Brien P, Dowsey M, Wallis J, Choong P, et al. How do people communicate about knee osteoarthritis? A discourse analysis. Pain Med. 2021;22(5). 10.1093/pm/pnab012. 10.1093/pm/pnab012 33502513

[37] Bunzli S, O’Brien BHealthSci P, Ayton D, Dowsey M, Gunn J, Choong P, et al. Misconceptions and the acceptance of evidence-based nonsurgical interventions for knee osteoarthritis. A qualitative study. Clin Orthop Relat Res. 2019;477(9):1975–83. 10.1097/corr.0000000000000784. 31192807 10.1097/CORR.0000000000000784 PMC7000096

[38] Arslan IG, van Berkel AC, Damen J, Bindels P, de Wilde M, Bierma-Zeinstra SMA, et al. Patterns of knee osteoarthritis management in general practice: a retrospective cohort study using electronic health records. BMC Prim Care. 2024;25(1):2. 10.1186/s12875-023-02198-z. 38166639 10.1186/s12875-023-02198-z PMC10759465

[39] Bunzli S, Taylor NF, O’Brien P, Wallis JA, Caneiro JP, Woodward-Kron R, et al. Broken Machines or active bodies? Part 2. How people talk about Osteoarthritis and why clinicians need to change the conversation. J Orthop Sports Phys Ther. 2023;53(6):325–30. 10.2519/jospt.2023.11880. 37259542 10.2519/jospt.2023.11880

[40] Haber T, Hall M, Dobson F, Lawford BJ, McManus F, Lamb KE, et al. Effects of hip pain diagnostic labels and their explanations on beliefs about hip pain and how to manage it: an online randomised controlled trial. J Orthop Sports Phys Ther. 2023;1–29. 10.2519/jospt.2023.11984. 10.2519/jospt.2023.11984 37795555

[41] Bunzli S, Taylor NF, O’Brien P, Wallis JA, Caneiro J, Woodward-Kron R et al. Broken Machines or Active Bodies? Part 3. Five Recommendations to Shift the Way Clinicians Communicate With People Who Are Seeking Care for Osteoarthritis. JOSPT, Inc. JOSPT, 1033 North Fairfax Street, Suite 304, Alexandria, VA … pp. 375 – 80. 10.2519/jospt.2023.11881 37383017

[42] Ringberg U, Fleten N, Førde OH. Examining the variation in GPs’ referral practice: a cross-sectional study of GPs’ reasons for referral. Br J Gen Pract. 2014;64(624):e426. 10.3399/bjgp14X680521. 24982495 10.3399/bjgp14X680521 PMC4073728

[43] Cottrell E, Foster NE, Porcheret M, Rathod T, Roddy E. GPs’ attitudes, beliefs and behaviours regarding exercise for chronic knee pain: a questionnaire survey. BMJ Open. 2017;7(6):e014999. 10.1136/bmjopen-2016-014999. 28624759 10.1136/bmjopen-2016-014999 PMC5541518

[44] Selten EM, Vriezekolk JE, Geenen R, van der Laan WH, van der Meulen-Dilling RG, Nijhof MW, et al. Reasons for treatment choices in knee and hip osteoarthritis: a qualitative study. Arthritis Care Res (Hoboken). 2016;68(9):1260–7. 10.1002/acr.22841. 26814831 10.1002/acr.22841

[45] Haskins R, Henderson JM, Bogduk N. Health professional consultation and use of conservative management strategies in patients with knee or hip osteoarthritis awaiting orthopaedic consultation. Aust J Prim Health. 2014;20(3):305–10. 10.1071/PY13064. 23863752 10.1071/PY13064

[46] Pacheco-Brousseau L, Charette M, Poitras S, Stacey D. Effectiveness of patient decision aids for total hip and knee arthroplasty decision-making: a systematic review. Osteoarthritis Cartilage. 2021;29(10):1399–411. 10.1016/j.joca.2021.07.006. 34302958 10.1016/j.joca.2021.07.006

[47] Lawford BJ, Bennell KL, Hall M, Egerton T, McManus F, Lamb KE, et al. Effect of Information Content and General Practitioner Recommendation to Exercise on treatment beliefs and intentions for knee osteoarthritis: an online Multi-arm Randomized Controlled Trial. ACR Open Rheumatol. 2023;5(1):17–27. 10.1002/acr2.11513. 36444919 10.1002/acr2.11513 PMC9837392

[48] Morton K, Beauchamp M, Prothero A, Joyce L, Saunders L, Spencer-Bowdage S, et al. The effectiveness of motivational interviewing for health behaviour change in primary care settings: a systematic review. Health Psychol Rev. 2015;9(2):205–23. 10.1080/17437199.2014.882006. 26209209 10.1080/17437199.2014.882006

[49] Gibbs AJ, Wallis JA, Taylor NF, Kemp JL, Barton CJ. Osteoarthritis management care pathways are complex and inefficient: a qualitative study of physiotherapist perspectives from specialised osteoarthritis services. Musculoskelet Care. 2022;20(4):860–872. 10.1002/msc.1638 10.1002/msc.1638 PMC10084427 35403316

[50] Briggs AM, Houlding E, Hinman RS, Desmond LA, Bennell KL, Darlow B, et al. Health professionals and students encounter multi-level barriers to implementing high-value osteoarthritis care: a multi-national study. Osteoarthr Cartil. 2019;27(5):788–804. 10.1016/j.joca.2018.12.024. 10.1016/j.joca.2018.12.024 30668988

[51] Ackerman IN, Skou ST, Roos EM, Barton CJ, Kemp JL, Crossley KM, et al. Implementing a national first-line management program for moderate-severe knee osteoarthritis in Australia: a budget impact analysis focusing on knee replacement avoidance. Osteoarthr Cartil Open. 2020;2(3):100070. 10.1016/j.ocarto.2020.100070. 36474677 10.1016/j.ocarto.2020.100070 PMC9718332

[52] Burn E, Murray DW, Hawker GA, Pinedo-Villanueva R, Prieto-Alhambra D. Lifetime risk of knee and hip replacement following a GP diagnosis of osteoarthritis: a real-world cohort study. Osteoarthritis Cartilage. 2019;27(11):1627–35. 10.1016/j.joca.2019.06.004. 31220608 10.1016/j.joca.2019.06.004

[53] McHugh GA, Campbell M, Luker KA. GP referral of patients with osteoarthritis for consideration of total joint replacement: a longitudinal study. Br J Gen Pract. 2011;61(589):e459. 10.3399/bjgp11X588420. 21801538 10.3399/bjgp11X588420 PMC3145529

[54] Marks D, Comans T, Bisset L, Scuffham PA. Substitution of doctors with physiotherapists in the management of common musculoskeletal disorders: a systematic review. Physiotherapy. 2017;103(4):341–51. 10.1016/j.physio.2016.11.006. 28801031 10.1016/j.physio.2016.11.006

[55] Desmeules F, Toliopoulos P, Roy JS, Woodhouse LJ, Lacelle M, Leroux M, et al. Validation of an advanced practice physiotherapy model of care in an orthopaedic outpatient clinic. BMC Musculoskelet Disord. 2013;14:162. 23656928 10.1186/1471-2474-14-162 PMC3658921

[56] Mazzei DR, Ademola A, Abbott JH, Sajobi T, Hildebrand K, Marshall DA. Are education, exercise and diet interventions a cost-effective treatment to manage hip and knee osteoarthritis? A systematic review. Osteoarthritis Cartilage. 2021;29(4):456–70. 10.1016/j.joca.2020.10.002. 33197558 10.1016/j.joca.2020.10.002

[57] Grønne DT, Roos EM, Ibsen R, Kjellberg J, Skou ST. Cost-effectiveness of an 8-week supervised education and exercise therapy programme for knee and hip osteoarthritis: a pre–post analysis of 16 255 patients participating in good life with osteoArthritis in Denmark (GLA: D). BMJ open. 2021;11(12):e049541. 34903537 10.1136/bmjopen-2021-049541 PMC8672017

[58] Dell’Isola A, Jonsson T, Ranstam J, Dahlberg LE, Ekvall Hansson E, Education. Home Exercise, and supervised Exercise for people with hip and knee osteoarthritis as part of a nationwide implementation program: data from the Better Management of patients with Osteoarthritis Registry. Arthritis Care Res (Hoboken). 2020;72(2):201–7. 10.1002/acr.24033. 31325229 10.1002/acr.24033

[59] Jönsson T, Eek F, Dell’Isola A, Dahlberg LE, Ekvall Hansson E. The Better Management of Patients with Osteoarthritis Program: outcomes after evidence-based education and exercise delivered nationwide in Sweden. PLoS ONE. 2019;14(9):e0222657. 31536554 10.1371/journal.pone.0222657 PMC6752869

[60] Wallis JA, Barton CJ, Ackerman IN, Sherwood J, Kemp JL, Young K, et al. A survey of patient and medical professional perspectives on implementing osteoarthritis management programs for hip and knee osteoarthritis. Musculoskelet Care. 2023;21(1):272–82. 10.1002/msc.1698. 10.1002/msc.1698 36101975

[61] The Royal Australian College of General Practitioners. Annual Report 2022-23. East Melbourne, VIC: RACGP; 2023.

[62] Gibbs A, Barton CJ, Taylor N, Kemp JL, Wallis J, Ezzat AM, GENERAL PRACTITIONERS STRUGGLE TO FACILITATE RECOMMENDED CARE FOR HIP AND KNEE OSTEOARTHRITIS DUE TO MULTI-LEVEL BARRIERS. : A QUALITATIVE STUDY [Poster Presentation]. Osteoarthritis and cartilage. 2024;32:S218. 10.1186/s12875-024-02658-0 PMC11657540 39702012

