# Supplementary material for: General practitioners experience multi-level barriers to implementing recommended care for hip and knee osteoarthritis: a qualitative study
Source: BMC Prim Care. 2024 Dec 19;25:423. doi: 10.1186/s12875-024-02658-0 (PMC11657540; doi:10.1186/s12875-024-02658-0)
Supplement: Supplementary file 1 — Supplementary Material 1: Interview topic guide [file 12875_2024_2658_MOESM1_ESM.docx]

**Topic Guide GP interviews**

1. **Current practice (to inform development of GP workshop and tool kit)**

How often do you see patients with knee or hip osteoarthritis in your practice?

Can you describe how you typically diagnose a patient with osteoarthritis?

*Prompt: What challenges do you have making a diagnosis? What is your confidence like in making a diagnosis? What role does imaging play?*

Please describe your typical management approach with these patients (i.e. order x-rays, ask about risk factors/lifestyle, refer to orthopedic surgeon or physiotherapy).

*Prompts: What referrals if any would you make? What factors do you consider e.g. lifestyle, BMI, pain severity, costs/income)?*

How confident do you feel managing these patients with osteoarthritis?

*Prompts: Share any challenges you find in managing these patients with osteoarthritis.*

What are your thoughts on the role of exercise therapy in managing people with osteoarthritis? What effect do you feel exercise has on/for people with osteoarthritis?

*Prompts: Do you prescribe your own exercises or physical activity to patients with osteoarthritis? What is your approach to exercise prescription?*

*Further Prompts if needed: do you refer patients with osteoarthritis to other health professionals for exercise?*

*What role do imaging results play in your decision making/views on exercise?*

*How safe/beneficial do you feel exercise is for people with osteoarthritis? What factors would make you more or less likely to recommend/refer for exercise?*

*If you know about it, what are your thoughts on the GLA:D program?*

*How could we raise awareness of GLAD/OAHKS*

What are your thoughts on the role of weight management in people with osteoarthritis?

What are your thoughts on the role of education and self-management in people with osteoarthritis?

What are your thoughts on the role of medication in people with osteoarthritis?

What are your thoughts on the role of injections in people with osteoarthritis?

What are your thoughts on the role of surgery for people with osteoarthritis? (e.g. Arthroscopic or TJR)?

What aspect of care (i.e. diagnosis, pain management, exercise) do you find most challenging in managing people with osteoarthritis?

How do guidelines inform your practice?

1. **Barriers to referral and non-surgical management of OA**

What do you feel makes it hard for patients to engage with non-operative management?

What would make it easier for patients to engage with non-operative management?

How do you decide when to refer a patient to physiotherapist led osteoarthritis services? (GLA:D And OAHKS?)

What, if any, benefits would you see for the patient in referring them to physiotherapist led services for osteoarthritis?

How confident do you feel in knowing where to refer people for osteoarthritis for further care?

*Prompt: When to refer patients? How to refer patents?*

What factors make it harder for you to refer people to physiotherapist led osteoarthritis services? (GLA:D And OAHKS?)

What would assist you in referring people to physiotherapist led services? (GLA:D and OAHKS) Other non-operative management?

What concerns, if any, do you have with referring people for physiotherapy and exercise? OAHKS?

*Prompt: Do you perceive any harms?*

Do you see it as part of your role as a GP to refer patients with osteoarthritis to physiotherapist-led services?

1. **GP Training, learning preferences and resource use (reported separately)**

Can you tell me about any PD training you have undertaken in OA management? How would you feel about taking further training in osteoarthritis management?

If you were wanting to learn more about a topic, what is your preferred method of learning (e.g. reading articles, podcasts, webinars, informal peer learning, topic champion within practice, conferences)? Where do you go for webinars/podcasts/how find out about them?

Please look at this table below and tell me your thoughts on the proposed interventions: (encourage to talk aloud as looking at it)

| **Components of the REFER Intervention** | | |
| --- | --- | --- |
| Workshop  (online or in person) | 2-hour GP-targeted osteoarthritis management workshop  Delivered by research team including physiotherapists, GP, and patient with OA | Content will be based on GP input and could include:  -Osteoarthritis background, e.g. prevalence, burden, co-morbidities, costs to health system and individuals  -Diagnosis  -Summary of guideline recommendations  -First line-care and supporting evidence  -Pharmacological (tablets or injections) evidence  -How and where to refer patients to first-line care (GLA:D or OAHKS)  -When to refer for surgical opinion  -Treatment costs, including physiotherapy, GLA:D, OAHKS, injections, private ortho  - Patient experiences of GP management and other non-surgical care |
| Referral template | Embedded referral template to physiotherapy-led services (OAHKS and GLA:D) within EMR | See example template |
| Interactive online osteoarthritis toolkit | Designed for GPs using input from GP interviews | Content will be based on GP input and could include:  -Osteoarthritis background, guideline recommendations, and evidence summaries  -How to refer, where can refer, costs of services  -Links to online (printable) patient resources  -Interactive aspects (e.g. video clips, quizzes, infographics) |
| Lunch time osteoarthritis booster sessions | 30-minute session, 4 months after initial workshop (online or in person) | Optional session including:  -Revision of workshop content  -patient outcomes from study so far |
| Marketing materials | Provide GPs with access to video clips, posters, brochures about first line care for osteoarthritis | See examples |

Questions

-What are your thoughts on these proposed interventions?
-Out of these proposed interventions, which do you feel would be most helpful to you in daily practice?
-What is the most important information you would like to know in a 2 hour workshop?
-What information would be helpful to you in an online toolkit?
-If you are providing patients with resources, would you prefer printed or online resources or a mix of both?

**Topic Guide GP interviews Modified**

1. **Current practice**

How often do you see patients with knee or hip osteoarthritis in your practice?

Can you describe how you typically diagnose and manage a patient with osteoarthritis?

*Prompt: What challenges do you have making a diagnosis? What is your confidence like in making a diagnosis? What role does imaging play?*

How confident do you feel managing these patients with osteoarthritis?

*Prompts: Share any challenges you find in managing these patients with osteoarthritis.*

What are your thoughts on the role of exercise therapy in managing people with osteoarthritis? What effect do you feel exercise has on/for people with osteoarthritis?

*Prompts: Do you prescribe your own exercises or physical activity to patients with osteoarthritis? What is your approach to exercise prescription?*

*Further Prompts if needed: do you refer patients with osteoarthritis to other health professionals for exercise?*

*What role do imaging results play in your decision making/views on exercise? How safe/beneficial do you feel exercise is for people with osteoarthritis? What factors would make you more or less likely to recommend/refer for exercise?*

What aspect of care (i.e. diagnosis, pain management, exercise) do you find most challenging in managing people with osteoarthritis?

How do guidelines inform your practice?

1. **Barriers to referral and non-surgical management of OA**

What do you feel makes it hard for patients to engage with non-operative management?

What would make it easier for patients to engage with non-operative management?

How do you decide when to refer a patient to physiotherapist led osteoarthritis services? (GLA:D And OAHKS?)

What do you know about GLAD? OAHKS?

*How could we raise awareness of GLAD/OAHKS?*

What, if any, benefits would you see for the patient in referring them to physiotherapist led services for osteoarthritis?

*Prompt-community physio AMP service*

How confident do you feel in knowing where to refer people for osteoarthritis for further care?

Prompt: When to refer patients? How to refer patents?

What factors make it harder for you to refer people to physiotherapist led osteoarthritis services? (GLA:D And OAHKS?)

What would assist you in referring people to physiotherapist led services? (GLA:D and OAHKS) Other non-operative management?

What concerns, if any, do you have with referring people for physiotherapy and exercise? OAHKS?

*Prompt: Do you perceive any harms?*

*Do you see it as part of your role as a GP to refer patients with osteoarthritis to physiotherapist-led services?*

1. **GP Training, learning preferences and resource use (reported separately)**

Can you tell me about any PD training you have undertaken in OA management? How would you feel about taking further training in osteoarthritis management?

If you were wanting to learn more about a topic, what is your preferred method of learning (e.g. reading articles, podcasts, webinars, informal peer learning, topic champion within practice, conferences)? Where do you go for webinars/podcasts/how find out about them?

Would CPD points be important to you in attending a workshop?

Please look at this table below and tell me your thoughts on the proposed interventions: (encourage to talk aloud as looking at it)

| **Components of the REFER Intervention** | | |
| --- | --- | --- |
| Workshop  (online or in person) | 2-hour GP-targeted osteoarthritis management workshop  Delivered by research team including physiotherapists, GP, and patient with OA | Content will be based on GP input and could include:  -Osteoarthritis background, e.g. prevalence, burden, co-morbidities, costs to health system and individuals  -Diagnosis  -Summary of guideline recommendations  -First line-care and supporting evidence  -Pharmacological (tablets or injections) evidence  -How and where to refer patients to first-line care (GLA:D or OAHKS)  -When to refer for surgical opinion  -Treatment costs, including physiotherapy, GLA:D, OAHKS, injections, private ortho  - Patient experiences of GP management and other non-surgical care |
| Referral template | Embedded referral template to physiotherapy-led services (OAHKS and GLA:D) within EMR | See example template |
| Interactive online osteoarthritis toolkit | Designed for GPs using input from GP interviews | Content will be based on GP input and could include:  -Osteoarthritis background, guideline recommendations, and evidence summaries  -How to refer, where can refer, costs of services  -Links to online (printable) patient resources  -Interactive aspects (e.g. video clips, quizzes, infographics) |
| Lunch time osteoarthritis booster sessions | 30-minute session, 4 months after initial workshop (online or in person) | Optional session including:  -Revision of workshop content  -patient outcomes from study so far |
| Marketing materials | Provide GPs with access to video clips, posters, brochures about first line care for osteoarthritis | See examples |

Questions

-What are your thoughts on these proposed interventions?
-Out of these proposed interventions, which do you feel would be most helpful to you in daily practice?
-What is the most important information you would like to know in a 2 hour workshop?
-What information would be helpful to you in an online toolkit?
-If you are providing patients with resources, would you prefer printed or online resources or a mix of both?
