# Supplementary material for: General practitioners experience multi-level barriers to implementing recommended care for hip and knee osteoarthritis: a qualitative study
Source: BMC Prim Care. 2024 Dec 19;25:423. doi: 10.1186/s12875-024-02658-0 (PMC11657540; doi:10.1186/s12875-024-02658-0)
Supplement: Supplementary file 2 — Supplementary Material 2: Supplementary material additional quotes. Supplementary Table 1: Additional Quotes Theme 1 GPs have good general knowledge of recommended care; and Supplementary Table 2: Additional Quotes Theme 2 General practitioners face challenges facilitating or directly providing evidence-based care [file 12875_2024_2658_MOESM2_ESM.docx]

| Table 1: Additional Quotes Theme 1 GPs have good general knowledge of recommended care | |
| --- | --- |
| Subtheme | Quote |
| Exercise and weight loss are important | *Almost every case [would refer on for exercise]. Because the way I see it is, one, even if the problem is acute for you, you need to not only help the current situation, but prevent it from worsening. And same, if you are looking at an operation at some stage, then you need to condition your muscles well enough to recover.* *(GP19)*  *Some of the patients, they actually improve really good with that… so basically, the combination of education and exercise, it works very good, yes, definitely. (GP21)*  *Actually, that’s the first step that I would do. So, to see a physiotherapist – I would explain, “Okay, you do some exercises, learn what to do, what not to do from a trained person,” and then, to give some more strength to the joint by strengthening the muscles around, so have some more flexibility about – so, those sorts of things, I say. Yeah. They do go there. They do exercises. (GP7)*  *Mainstay of the treatment is exercise, because after knee or hip replacement, we need that much more than what they have in the normal life. Anyway, they need to learn that . . . they need more doing exercise to get that range of motion they are expecting from before surgery. (GP8)*  *Exercise and weight loss, the two are connected. Diet, exercise, weight loss, for me, are everything I do, whether it's osteoarthritis, or diabetes, or whatever disease I'm dealing with, I do a lot of weight loss, weight management. And in that weight management, diet, and exercise are the mainstay of management, of course. So I'm heavily into it. The benefits are unchallenged, we know how effective it is, so definitely they're the mainstay of my management plans. (GP24)*  *[Exercise is] really important. . . But osteoarthritis, any movement is important. Even hydrotherapy in my opinion is really – as long as you keep moving your joint, that’s necessary . . . If someone has really bad osteoarthritis, and they cannot weight-bear, they’re in pain, then trying to do hydrotherapy or any movement is better than no movement. (GP13)*  *But there are a few things we can do. So number one, trying to do the regular movements, regular activities – not vigorous exercise, but just simple walking and keep moving. The joints should be moving. (GP13)*  *So, balance exercise, dietary management, sometimes the supplements, I do that. And I keep following up with them. And physio is really important, so I send them to a physio. . . Now, I am very big in physio. So I know there are other allied health there. There are some manipulative therapists there as well, but I send my patients first line to physio. So I tell them always just keep moving, the normal movement of any joint to keep moving. You don’t have to forcefully try and do things, but just your normal movement. (GP13)*  *I think it’s [exercise is] really important. It’s good, all those things, like weight reduction, exercises to strengthen muscles around the knee, supporting knee, and just maintain mobility, I think is really good, rather than if people don’t tend to use it, they tend to stiffen up and get more pain, and you have a bit of downhill slide, really. So I think it’s really important. (GP16)*  *Definitely very important. The more they exercise and build their muscle strength, the longer they can go without having an operation, so definitely important. (GP11)*  *I think there are adjuncts and not things that are gonna be the main stay. I think exercise and weight loss are the main things. (GP22)*  *[I] think, yeah, they’re [exercise and weight loss] very important . . . So, and they haven’t been exercising at all, so it’s sort of – it feeds back into their weight as well, so if we can get their weight down with some exercise, it will help with the symptoms of the OA, but it will also help if they do end up needing surgery. So, I do try and push the exercise a fair bit. (GP12)*  *So I often say to them, it’s about, when you start getting pain or arthritis within a joint, often people stop using it so it gets weak, so what we need to do is improve the strength of the muscle around the joint which will help with the pain . . . So really the exercise, weight loss and then pain management . . . So that’s where you really need to be clear that the exercise and the weight loss is really the key. (GP17)*  *I mean there’s a role for exercise in managing every patient that I see (GP1)*  *That’s number one. It’s education and then keep active, moving, exercise, water therapy, hydrotherapy, physiotherapy, stretch exercise. I mean, basically, osteoarthritis people don’t move enough. (GP18)*  *You can probably go for that phrase, “use it or lose it”. I think if you just don’t do much exercise, things are gonna deteriorate around with joints, and so, I guess strength – exercise strengthening of the muscles around the joint can take pressure off it and help reduce pain. (GP6)*  *So putting on weight, definitely will produce pain, and if they can reduce 7% of the weight, it works as a Nurofen. (GP8)*  *It’s another cornerstone for those that are obese. There’s clear benefits in the literature for knee osteoarthritis, and though the benefits don’t seem to be as well-established for hips, but there seems to be perhaps some biochemical factors involved also that might help osteoarthritis of other areas, but the effect is strongest in knees. (GP22)*  *Yeah, [weight management has a] big role. Yeah. Big role and it’s funny because patients seem to know that their weight is impacting on their joints and their joint pain and their arthritis and they want a quick fix to the weight loss in order to fix their knees. So, we do a lot of weight loss medicine here at this practice, from traditional to alternative approaches. But I would say that the majority of my patients, with particularly OA of the knee, do recognise that it’s due – it’s contributed to by their weight. (GP12)*  *I definitely think weight management can be really useful, . . . I do think it’s definitely got a role, particularly in patients where they’re very overweight, where I think it’s inhibiting their exercise, it’s inhibiting all those other things that are gonna go along with it . . . So, I definitely think it’s an important part of it in a plan (GP3)*  *Everybody should manage their weight with or without osteoarthritis, absolutely (GP5)*  *Definitely, it’s very important. I do try to work on that, try to get them to lose weight because usually it’s obesity that leads to – especially with the knees and hips and all that. It’s due to the extra weight that they’re carrying that results in the joint space narrowing and then arthritis as well. (GP11)*  *If somebody’s very overweight, then we’ll be wanting to focus on trying to target that, to try and just take the weight off their sort of pressure joints. And that can have a huge benefit (GP6)*  *My first option will generally be a program through a physiotherapist or things that might help with pain, whether that’d be other allied health, like massage or go to a pain specialists for it. The GLA:D programs through physios is one of the first things I'll give a go. (GP10)* |
| Uncertainty around exercise (benefits, types, prescribing) | *Cause I’m a registrar and I’m only in a clinic for six months, I probably don’t have that longitudinal experience. I mean I think it works, I guess, when you don’t have that longitudinal experience . . . but I don’t know if those programs work and – I don’t know, group exercises programs or something. I don’t really know (GP23)*  *I will tell them, I will leave it, that more of these to the physio. . . . If they have YouTube, then I'm telling “You go with a good one, good video.” That’s why I'm keeping the videos in my folders, I'm giving them, show them, “Look, take a video, from that go and look at the YouTube, start doing the video. And if you need more strength like an athlete or things, go and see a good physio, a good physio to show you how to do the exercise, not just harming more, you need to do properly.” That's why I know that if you're not doing properly, they will harm. (GP8)*  *Well at what point would I tell the 50-year-old whose come and say, “Oh God, I'm getting old, 50,” and you're like, “I am old. I’m 49, so you can’t be older,” and am gonna send every one of them off to the physio? Probably not. I’ll just say, “Just make sure you stay active.” . . .* *Maybe I’ve not had the confidence ‘cause I can’t teach the exercise– I don’t think you should dabble. I don’t think people should dabble.* *So if I don’t feel like I have the confidence to teach them properly, then I won’t. (GP5)*  *So, I probably don’t push it that hard though, I must say, probably not something I emphasise in consultations. I’ll probably leave that to the physio to sort out. (GP6)*  *You get someone early, I think it can be really useful and definitely I’m seeing that in my personal life, [and] in patients. If they get involved in exercise with mild symptoms, I think it can be really useful and prevent need to do anything else. (GP3)* |
| Challenges of weight management | *I guess there’s that cycle of readiness, like whether they’re ready to make a change or not. Some people I’ve been telling for years, “You really need to lose weight,” and they just aren’t interested. I’m just not gonna waste the time. I’ll mention it but I’m not gonna put any effort into it. So it’s locus, like you can’t until they’re ready. (GP5)*  *Yes, I'm offering them to see dietitian, but this is not that good (GP8)*  *I tell you, it’s very, very difficult. It’s extremely difficult. Because of the knee and hip pain, they can’t exercise. Because they can’t exercise, they put on weight. So it’s a vicious cycle. It’s a very, very difficult thing to break the cycle and I personally struggle. There are a number of patients struggling – absolutely difficult, so up to the point sometimes there’s no clear solution for them. A lot of times, these people who are obese, who’s got severe osteoarthritis and have got other medical conditions, maybe heart failure, maybe some lung disease, and they’re not surgical candidates either . . . So it’s a very challenging situation . . . Weight management is difficult. If anyone has a magic pill, that will-I need to give to my patients . . . Once you mention it, they say, “Oh, no, doctor, I don’t eat that much. Believe me, I don’t eat that much.” So then I tell you, “It’s not that much you’re eating. Even if you eat a pea, your body’s metabolism is too slow and it will make too much out of it.” So then you’re trying to educate them. So, weight management, going back to your initial question, it’s very, very difficult. I face that almost every day of my career. (GP13)*  *Most of the people who’ve got osteoarthritis, they come to us when they are not able to walk properly or mobilise properly and they’ve accumulated a fair bit of weight that is actually compounding to their current illness. And then to tell them that you need to lose weight and you’ll get benefitted – I mean, generally, they say “Look, we can’t move because of the bad pain.” So, I have to tell them again and again . . . What I find the most challenging part is to tell them that “Look, you need to lose weight.” That’s the most difficult part because it’s not easy and then, of course, I do hear from them, “It’s easy for you to say than for us to do.” (GP2)*  *But a lot of them are complaining like they can’t do anything about their weight because their knees are so sore and it’s really hard to rationalise with them that, you know, you just keep going. (GP12)*  *It is harder to get them to consider the benefits of nutritional assessment and weight loss but part of that is just the overall difficulty in terms of affecting weight loss. There’s a huge difficulty which most people who are obese have tried to tackle previously. It’s really hard. (GP22)*  *Obviously, there’s a lot of barriers to people being able to lose weight, all sorts of mental and lifestyle and all sorts of other issues, motivation issues and things, and emotional eating and everything. So, it’s not – whilst it might be easy theoretically, putting it to practice can be really difficult. (GP6)*  *So weight management, but I think is a whole other topic, and something that’s not particularly successfully approached or managed for the vast majority. (GP14)* |
| ‘Holistic’ approach to weight | *So, I definitely think [weight management] it’s an important part of it in a plan, but I would always be careful not to make that the only recommend – or I think give a context of where it’s important and not just blame it all on someone’s weight. (GP3)*  *So as far as I’m aware, the evidence is good, that losing weight is good and possible, but it’s obviously a very complex area, as in like when you’re addressing it with patients. So usually when I’m discussing movement and exercise with patients, I try to emphasise that weight loss is good but even if you don’t lose weight, movement is still good. I might mention weight loss in passing but I try to keep the focus on exercise and movement just ‘cause I think that’s more likely to be successful. (GP23)*  *Obviously we know that managing your weight is quite important when it comes to arthritis but, I think that that is something that you can approach in a different way as oppose to just saying, “Look, we know that if you lost some weight, it probably would help,” instead, I think we can try and focus more on getting the person moving. . . . I talk to people less about weight management, and I talk more about healthy behaviours. Because being fat isn’t a modifiable behaviour but how much you move is something we can modify. (GP1)* |
| ‘Reduce joint load’ approach to weight management | *If somebody’s very overweight, then we’ll be wanting to focus on trying to target that, to try and just take the weight off their sort of pressure joints . . . I’m quite happy to try dietary approaches and then maybe medication if it’s necessary. And then, some people even – bariatric surgery. (GP6)*  *I do try to work on that [weight loss], try to get them to lose weight because usually it’s obesity that leads to – especially with the knees and hips and all that. It’s due to the extra weight that they’re carrying that results in the joint space narrowing and then arthritis as well. I do manage them myself most of the time. Of course, if they are morbidly obese, then I might refer them to obesity clinics or bariatric surgery things like that. It depends on how obese they are. So, if it’s mild-to-moderate obese, I will treat them. If they’re morbidly obese, then I’ll try to refer them as well. And we use medication as well in terms of weight loss as well. (GP11)*  *Oh yeah. Everybody should manage their weight with or without osteoarthritis, absolutely, less load on the joints. It probably is something I’d mention, straight off, that keeping your weight as low as possible to take some weight off your joints (GP5)* |
| Imaging and patient expectations | *I have to say I probably do it for most people, I think there’s definitely an expectation. So whether it’s just, I just don’t really think about it that hard and I just do an x-ray ‘cause everyone expects to have one done and I figure it’s a relatively cheap and minimal radiation so yeah, I’d usually do an x-ray. (GP23)*  *Look, I do radiology, x-rays, probably earlier than I really need to ‘cause sometimes patients like to know what it is that they’ve got, absolutely. (GP17)*  *The challenge nowadays is people don’t want to be treated. People just love to be investigated. . . People just come with a set mind of “I want MRI” and they have no symptoms to require MRI, or “My friend told me that” or “My uncle said this,” or he went to the hospital and the hospital done a scan from head to toe. I mean, this is the perception of the people (GP18)*  *. . . think that [referring for x-ray] helps with explaining to people in advance as to why I’m not doing an MRI scan because, inevitably, some of them are going to ask for it potentially, “Don’t you think I need an MRI to see what’s going on in there?” (GP1)*  *The patient expects if they come in and they’ve got hip or knee pain . . . that’s their expectation, they’re coming in thinking they’ve got that wanting me to confirm it with an x-ray. (GP14)* |
| Imaging undertaken for confirmation | *Sometimes I use imaging to confirm it or show the patient that it's just arthritis and not something else. So really is just reinforcing that it's arthritis rather than other MSK issues (GP25)*  *Well, sometimes an x-ray is a good idea, just to make sure that we can see what’s going on in the joint, and give us an idea of that. (GP1)*  *I can diagnose without imaging, but for confirmation, documentation, and for progress, I will do a simple plain x-ray as a standard for all patients. (GP8)*  *I would say “on the way it’s developed slowly overtime, that’s probably osteoarthritis. We might do an x-ray to see, look where you’re at, because down the track, you might need to have something done like surgery way down the track, so let’s do an x-ray just to get a bit of an idea what’s it’s looking like.” (GP5)*  *I’ve usually got a pretty good idea whether someone’s got knee arthritis and it’s useful also to look at the severity of the of the arthritis of the joint, so normally just plain x-rays for the knee and hip too (GP6)*  *Often, it may be something that I might sort of be suspecting clinically, and I may get an x-ray to help the diagnosis, depending on if I’m a bit uncertain, or if I think it’s pretty straightforward, I may not. I may just diagnose it clinically, but I think if I’ve got any concerns about the diagnosis, you know, I usually would do and x-ray as it’s, you know, I feel like it’s pretty low intervention or low risk, and sometimes might provide helpful information or at least rule out other causes. (GP3)*  *And with the hip, I’m probably more likely to do an x-ray probably because often they’ve got symptoms [pauses]– because it’s the groin pain but they might also have pain to the trochanteric bursa, I’ll say like, “what if there’s some bursitis, let’s do an x-ray and ultrasound,” and sometimes I might sound dumb but you're not sure if there’s an inguinal hernia if it’s in the groins, so I’ll just wanna confirm it. So I’m probably more likely to do an x-ray with the hip. (GP5)* |
| Imaging not required for diagnosis | *If I felt like it was really early days, I wouldn't. Sometimes I would get a baseline x-ray if I'm thinking like, okay, in ten years, this person may need a total knee replacement or hip replacement, then you'd want that baseline image but, of course, you try not to x-ray people because of the radiation risk. (GP24)*  *Not really, no. It’s not a blanket – the request, no. I don’t do investigations unless there is a clear reason for that. So, I mean, whenever I request investigations, just for the sake of diagnosis, I don’t do this, but if I request an investigation and there must be a reason for that. The reason is either to establish a diagnosis and then there might be something we can do about it or to evaluate the degree of the disease. (GP13)*  *Not with everyone, particularly not if I don’t think it’s very severe. So, if it’s not something that’s really stopping them or someone that I first see – like if it’s not someone that’s gonna need a surgical review soon, I generally do a clinical diagnosis. (GP12)*  *Generally, I won't image unless I’m thinking that the person maybe has an atypical sort of presentation, or perhaps that they’re getting towards the stage that they’re failing conservative management and that they may need surgery. They would be pretty much the only times that I would routinely do imaging. (GP22)* |
| Imaging is required | *First of all, imaging, to establish how bad the disease is (GP15)*  *I do generally organise weightbearing x-rays for both knee and the hip if I am thinking that this is osteoarthritis I’m dealing with and – yeah. So, there will be, basically, the history examination and the x-ray findings. (GP2)* |
| Views on medication -paracetamol and NSAIDs | *Panadol, especially Panadol extended release, we usually do. I know it’s questionable whether it works or not but I mean it’s cheap and it doesn’t really interact with anything, so I don’t think it’s harmful. (GP23)*  *I like to keep it simple with just paracetamol (GP24)*  *It is sort of a reflex action. Once we have osteoarthritis, we tend to start something like Panadol or Panadol Osteo. You tend not to give these people, so NSAIDs or non-steroidal anti-inflammatory medications. In elderly, you don’t wanna give it to them, because that will affect their liver, their kidney, and that will make a hole in their stomach, and they got multiple other interactions with other medications. You cannot – so you don’t have much choice. You go for pain management only.* *(GP13)*  *I start with Paracetamol then you go on to nonsteroidal, but then I’ll try and use nonsteroidal as less frequently as possible because this is gonna be a long-term problem (GP11)*  *Although the evidence is pushing against paracetamol, I think that’s certainly still got a role. (GP22)*  *Panadol, simple analgesia, it’s like lollies. It doesn’t work most of the time. It does have a placebo effect in some but – well, anti-inflammatory really works, they are beneficial but then again there is a limitation. We can’t keep on giving them an anti-inflammatory. (GP2)*  *If it’s an absolutely quiet joint, then I suggest using Paracetamol. But occasionally, anti-inflammatories if that doesn’t work. (GP17)*  *Anti-inflammatories all depend on, obviously, comorbidities. People seem to find them relatively helpful sometimes, so that’s either on as-needed basis or as a regular daily medication, depending on what people are after, and that’s pretty much all (GP23)*  *I’d like to start with Panadol, I guess, Panadol Osteo, something like that, regularly, and then move up along to a non-steroidal, depending on what the person’s other medical issues are, lots of people have got risk factors for chronic kidney disease, gastric ulcers, things like that. (GP1)*  *NSAID are very effective but have a lot of downsides in terms of side effect profile, but still it can be very helpful in some cases. (GP22)*  *Anti-inflammatories can be . . . helpful for some people but they’re contraindicated in quite a few people anyway who are getting into that age group where there are relative contraindications. (GP16)*  *People who are taking long-term anti-inflammatory, like Celebrex, Mobic, Naproxen, they’re not very good for long-term . . . maybe Panadol Osteo or something like that which is not that dangerous compared to anti-inflammatories (GP21)*  *Generally I give anti-inflammatories a go just to see if there is an inflammatory component, and if there’s not, get them off it, ‘cause they’ve got side effects. So, absolutely, just give it a go and see what difference it makes, and Panadol if it’s for pain. (GP5)*  *In the acute pain, I definitely prescribe anti-inflammatory and rather than have painkiller. Anti-inflammatory because inflammation is background to knee. So I'm prescribing the anti-inflammatory. (GP8)*  *Osteomol or Panadol Osteo, and then you accelerate another if need for SNRI, any of the anti-inflammatory, COX-2 or nonsteroidal, or mindful of check your renal function and be sure that they’re not having a problem with renal or triple whammy if they are on hypertension medication or diabetes, we don’t want to upset their kidney. (GP18)*  *I usually would suggest some simple analgesia like Panadol or Panadol Osteo. And then if it was more severe, I may trial an anti-inflammatory. I’m always a bit cautious often in that age group, you know, if there’s cardiovascular risk factors, it can be a little bit more concern. You know, I get some patients who have clear risk factors of being on anti-inflammatories, but really wanna take them, and it’s always a bit stressful. (GP3)* |
| Views on medication-opioids | *Opiate’s are best avoided. (GP22)*  *So I’m very, very reluctant in the community, relatively younger people to give them opiates for pain management. And I don’t think it is a good practice to give them opiates unless there is a clear reason. (GP13)*  *I’m not a very big fan of giving opioids because I know there’s a long-term risk of addiction, plus also, some of the time, I have had instances where at the end of – when a patient wasn’t responding so I actually had to give them a longer-acting opioid and they actually had a bit of nausea and vomiting. (GP2)*  *I never prescribe opioids, just to put that out there. Obviously, that’s fraught with so many issues and it’s not indicated but, unfortunately, I think it is still probably done in some people’s practice. (GP1)*  *I have some older patients who’ve been started by other GPs on opiates . . . not keen, not happy. (GP5)*  *I'm pretty strict about not doing any opiates for any chronic pain including osteoarthritis. I explain how it’s not helpful in neural pathways and all that type of stuff (GP23)*  *I would rarely ever prescribe opiate medications and I guess I, in some ways, look in the patient population I have, this doesn’t happen often, but I don’t think I would ever really want to – I can think of maybe once or twice I would have started someone on an opiate once, I think. I’m pretty reluctant to ever start someone on it for something like these where it’s gonna be a long-term issue because I just think there’s nowhere to go with that. Once you’re on it, it’s gonna be really hard to come off it and they can be so addictive… But I just think it’s so dangerous, so personally, I’d be really reluctant prescribe anything stronger than a Nurofen. I might seem harsh, but I just think that it’s not – I just don’t think it has much role – I don’t think there’s much evidence for having a role in osteoarthritis anyway. (GP3)* |
| Challenging to manage severe pain | *By and large, I don’t know if it [role of medication in managing OA] has a great deal, really, to be honest. (GP16)*  *if someone is waitlisted for surgery and they’re waiting to be seen by a surgeon, and for someone reason, it is getting delayed, and then you have to manage their pain, and there is a limit. There is an endpoint for that. Yes, that person – yes, just be generous. Be kind for them to make sure symptoms are controlled . . . So, if a normal person and they have osteoarthritis and the pain is related to chronic condition, you better be very careful in giving them stronger pain medicine. (GP13)*  *Medication role is really mainly with pain control and it’s certainly a very imperfect management strategy (GP22)*  *I mean, it doesn’t fix the real problem. I mean, yes, it is more like a numbing thing but it doesn’t really get rid of the original problem or doesn’t actually help them in improving their health otherwise. (GP2)*  *[I] try not to use anything really stronger than a nonsteroidal or a paracetamol. Occasionally, when patients are waiting for surgery, yes I will give them – I don’t let them suffer, but I try not to use opioids or opioid equivalents for arthritic pain unless they are waiting and in a severe category. (GP12)*  *I have some very, very old patients that I’ve started on opiates because their pain is just unbearable and they’re in their 90s, and they’re really seriously just got very little function left and they’re just waiting out their day, I’ll give the opiates because they can’t sleep or they can’t focus on anything else, but I do go down that rabbit hole sometimes but I don’t think it makes a huge difference. (GP5)*  *As it gets more severe – I think it’s quite difficult to find medication which really does, which works really well for people with arthritis, and probably use it less now than I used to do, but – yeah. (GP6)* |
| Views on injections | *One of my patients, she just recently had a cortisone injection and she was saying that she was feeling a lot better. I don’t know how long it will work, but some of them, they do – yeah. When it comes to having injection, I don’t have a plus and minus for it, because my understanding is for some, they work, yes. And for some, they don’t work at all. I suppose if it’s coming through a very severe osteoarthritis where they actually have bone-on-bone, that the injection does not work much. But then, again, if it is not as bad, it’s more like mild-to-moderate, it might give them some benefit. But then again, there’s a limit to how many times you can offer them injections. (GP2)*  *I do prolotherapy, I do PRP for all sorts of condition, but knee is very common. (GP21)*  *If we need it, if there is bulging and problem pain, we have to give [cortisone injection], but otherwise, I'm not favouring. I’m not in favour of that. (GP8)*  *I think, my understanding is, obviously, they [cortisone injections] are just a bit of a local anti-inflammatory, so they’re effective in taking away pain, but you can’t keep on doing it. I don’t know if there’s a specific limit but I know you just shouldn’t keep on doing it for years, so then it’s important to try to do it so that they get the injection and then immediately get the physio afterwards so that you’re maximising the strengthening and the exercise you can get done whilst you’re in that pain-free stage. It is not always that easy to coordinate. That’s my understanding of how it can be done. (GP23)*  *A long, long time ago, I used to do injections pretty regularly, but as the literature changed about evidence saying that they may have more harmful effects, a rule of thumb that I stick to is no more than two injections per joint per life. That’s just training from, I used to do a lot of surgery previously, so we know that's not the best way to go and again, more like a Band-Aid than a management plan, or a strategy, or treatment. (GP24)*  *Okay, steroid injection – they say two or three and then you’re out, something like that. To tell you the truth, I do refer them to ultrasound-guided steroid and local anaesthetic injections. I do refer them. That, again, depending on the patient and their condition and every individual. For an example, if a nursing home patient sitting in the wheelchair and they just move from bed to the toilet and then even that is affecting them badly on the knee, yes – and you’ve tried all the patches or the – externally you can use some creams. And in my opinion, most of the external agents are placebo, for an example, Voltaren gel or other things. But it helps for some elderly patients. Anyway, you tried all those things and still the patient is suffering. That is a good candidate for you to take them to inject something in the joint. That helps, and again, wear off very quickly. So injectable – the one I’m using is a steroid and local anaesthetic mix. I never do it myself alone, but I always send to radiology to do ultrasound-guided. Now, PRP injection, I don’t know where I stand, where is my head around it. I don’t know. I have to discover myself. I’m not with it. I’m not against it either. (GP13)*  *In terms of injectables, it depends who you talk to. There’s huge differences in the literature. I mean cortisone injections were the main stay of injectables. Now, there’s quite a bit of evidence in the literature perhaps that it’s not a good idea, certainly not to use repeated injections . . . I think still a role for aspiration. I had a lady today with long-term issues with a swollen knee and her knee was too painful for her to do her exercises, so we agreed to try that, a successful aspiration and then injection of corticosteroid but I don’t do a lot of them. I try and use them judicially. Also sometimes I will use prolotherapy, for injection of trigger points and perhaps for tender points around the joint line and around the pes, and distal medial quadriceps, and that can be quite helpful as an adjunct in pain relief, but there are range of other things that people are using, from glucosamine, glycosaminoglycans, hyaluronans, PRP, and there’s evidence for and against in the literature. I think there are adjuncts and not things that are gonna be the main stay. I think exercise and weight loss are the main things. (GP22)*  *Yeah, for patients that have Eufflexa . . . they’ve had good response. Some patients have had – I’m quite open to injections. Some patients have had some cortisone that’s given them relief for a number of months, which is great, because then they can actually do some activity. I do PRP occasionally on knees. I did a study on PRP for my masters and it showed an 84% increase in quality of life symptoms, so I don’t know whether that was placebo or actually from the PRP, but patients do seem to get a benefit from it. Yeah. So, look, I’m open to injectables. I will inject knees and shoulders and some ankles myself. I mean, I can do a hip without guidance but I prefer it done under guidance. I haven’t done a hip for years. So most of the injecting now is referred, yeah, that I’m open to it. (GP12)*  *Look, I’ve had people when they’re going on overseas for a holiday or whatever. I was actually giving them a steroid injection so they can do what they needed to do. Certainly in acute rheumatoid conditions, I’ve used steroid injections. They give initial benefit, but at six, nine months, there’s really no difference between those who went conservative as oppose to those who had a steroid injection. Hips again, if the pain is really becoming unmanageable and they need to do something, I will sometimes inject the hip while they’re deciding which surgeon who they’re going to see, but I know surgeons don’t like it within at least three to four months of them seeing it, so you’ve got to be a bit careful. (GP17)*  *Again, it’s not so much for arthritis. I know that some of the specialist likes to use things like Synvisc – I’m not even sure what that is, to be honest with you. I don’t do injections. I don’t inject joints myself and I don’t routinely refer – I’m just thinking about knees now. I don’t really get people like to have their knees injected with steroids. So I don’t do them myself, so it’s probably outside my limitations as a GP but certainly I’ve had people where a specialist might have recommended it but I don’t think those patients went through with it. (GP1)*  *I know that sometimes you see injections of steroid recommended. I don’t refer people for them myself. The occasions when I’ve seen it recommended sometimes by the orthopaedic people, and sometimes by rheumatologist, perhaps if they think if there’s an inflammatory component, but by and large, it’s not something that I’ve ever initiated myself. (GP16)*  *I think I forget they exist. It’s funny ‘cause we were taught as GP registrars, I think I did in the early day, inject a few knees and possibly shoulders, from memory. That’s funny, uh isn’t that interesting. I think if they’re at that point, I usually send them to an orthopaedic surgeon. (GP5)*  *You can’t say injection for everyone or you can’t say injection is not useful. Injection has a role – you are not denying that. I mean, this new injection which I think is Euflexxa, which is sodium hyaluronate, intra [Articular] injection and some of the radiologist love it and so you build up networking of radiologist who are experienced in joint injection, non-steroid based, something like Euflexxa or – Some of them are expensive, some of them affordable and some of them are happy to do it on a bulk billing. If the patient want to do PRP this is another area of treatment. (GP18)*  *I think they can be quite a useful thing to give yourself or to give someone a few months pain relief. So, if someone, say, wants to put off an operation, or is not fit for an operation, or – I think it’s perfectly reasonable approach to go with. But usually wouldn’t do it for somebody with mild arthritis, they have to have quite an advanced arthritis, and usually with effusions in the joint, ‘cause otherwise, I find this can be a bit tricky to get the right spot and I think probably less likely to see benefit as well. (GP6)*  *I haven’t had personally good experience with it [HLA or PRP]. I know that some of the research isn’t too bad, but I’ve never seen anyone benefit from any of those injections. So, that’s kind of skewed me away from them. So, I know sometimes when I refer to a specialist, they’ll organise them for patients, but I’ve yet to see anyone benefit from any of them. (GP6)*  *Um, yeah, I think it could be useful. I might admit this is something where probably my knowledge is a bit shaky. I definitely think it can have roles. Often my patients refer, just for instance, shoulder issues for a steroid injection. I haven’t done this many for osteoarthritis. My understanding is there’s a limit to how often you can do that or how many times since. So I guess, I would probably – I think it has a role, but I guess it’s probably not what you wanna jump to the first line, ‘cause you don’t really have anywhere to go if it doesn’t work, or it works and then it gets worse again. (GP3)* |
| Views on surgery | *Surgery is – I mean, if all else fails then you end up needing surgery, so surgery is probably the last resort. So, we’ll try and work out everything else as possible to see if that will help them and then if not, then, yeah, surgery. (GP11)*  *And then, of course, the last option is then we need to go for surgical procedure, and then I will refer to the surgeon . . . Surgery is not a magical option. It does not magically improve everything. But then again, at the end of the day, if say, for example, the osteoarthritis is actually too bad and it’s not responding, and it’s actually quite disabling for them or their routine activity, especially when they are independent or living by themselves then I would prefer we go for surgery . . . I’d really them to try your best for the non-operative because the longer you keep your own joint is the best that you can have and obviously, because in telling them about the most surgery, that prosthetic can last for such and such period of time. So if you are able to keep yourself longer, then it’s better for you. (GP2)*  *And a lot of them think that a knee replacement or hip replacement is the best thing since sliced bread and will make everything go away, and I point out to them, “There is nothing as good as their own joint when it comes to function and so on.” And really what we’re talking about is pain management. So they can’t think a joint replacement is going to be purely fantastic and then they’ll have an absolutely normal knee back. It doesn’t quite work that way . . . Look, absolutely great for people who need it . . . But I certainly don’t push early intervention. (GP17)*  *Everything has a time and place, so for some patients, we're always thinking about quality of life, so if their quality of life is terrible and their quality of life is probably going to significantly improve after surgery, I would recommend it, but if their quality of life is pretty decent and there's possibly high risks involved with the surgery, and possibly even more restricted movements or surgical complications then me and, obviously, all the surgeons would tell them to hold off as long as possible. (GP24)*  *Well, it’s end-stage, really . . . I get a lot of people with knee surgery get improvement on level of pain and function but, certainly there are a few more people who aren’t helped as much as they might have expected to be helped by the surgery but it’s generally only recommended when they’re at a very advanced stage of the osteoarthritis, really that they would be undergoing that anyway. (GP16)*  *So obviously sometimes, there’s a real role for it. I guess by the time I’m not managing them, by the time they’re really using an aid of some sort, and we’ve probably tried physio as well, they’re off to orthopaedic surgeons and then they decide. And my understanding is I do it once you can’t bear it anymore because it will never feel like it did, so you have to be willing to risk it. (GP5)*  *This is the end of the track. I mean, it’s not number one. (GP18)*  *I think generally, most surgeons would seem to prefer to put it off as long as possible ‘cause I think the joints only last so long, and then the redo is quite complex and difficult. So, yeah, I think in the right patients, it can be a really good option. (GP6)*  *It’s probably something that really you want to avoid. Having said that, I think there’s definitely some people who do get good benefit from joint replacement, like, there’s obviously people who have a joint replacement that really need it, and it does keeps them a better quality of life post the operation, but I guess, it’s a pretty – it’s not necessarily a low risk operation for some of these patients . . . I definitely don’t think it would be my first line. “Oh, you better see a surgeon, you’ve got osteoarthritis.” That would never be my first line approach. (GP3)* |

| Table 2: Additional Quotes Theme 2 General practitioners face challenges facilitating or directly providing evidence-based care | | |
| --- | --- | --- |
| Level | Subtheme | Quote |
| GP level | Challenging to address lack of motivation to exercise | *When you don’t have that longitudinal experience and it can be hard to convince people to try [exercise] when they are in pain. (GP23)*  *That thing I struggle with is getting patient to get motivated, I wouldn’t say I have a great solution to them (GP3)*  *A lot of patients want surgery and physio doesn’t cut it so, occasionally, I’ve even had to refer patients to surgeons so that the surgeon can tell them to go to the physio, because it seems to come better going from them. (GP12)*  *But a lot of the challenging thing is people said, after physio, after all the treatment, they don’t feel the immediate relief. That's very hard to call, not so easy to achieve. Like if you got a bacterial infection, antibiotics can fix in a few days, virus infection, rest or supportive treatment, or antibiotic treatment a couple of weeks. So we can't achieve obvious results straightaway. It's a long journey (GP20)*  *But certainly, that’s a challenge, what’s the patient’s motivation and are they motivated as well about, help seeking more than just coming to see me because I can only bring them so far on the journey (GP1)*  *Motivation – I’ve got one guy at the moment who’s got osteoarthritis with his knees and he’s a young builder and he’s got massive quads wasting, but he’s – I keep trying to get him to see the physio, but he just doesn’t want to go. (GP6)* |
|  | Lack of time in consultations | *Some of the physios, they have that brochures which have helped for them to understand what the program is about, because if you tell them, they may not have a clear idea, especially in a GP practice with a limited time. So I leave it to those physios who are really good and who are actually doing the program, and then they call the patient and they make them understand and that makes a big difference. (GP21)*  *Everything is too long, assess everything. I got no such time to do so. (GP20)*  *And sometimes you feel like in general practice, what we do is we look after patients but also massive paperwork, we have to write everything. And even there was care plans. You spend a lot of time – the EPC [care plan] – you have to spend as time based – you have to spend at least half an hour with the patient. So it’s not easy. (GP13)*  *And then it is always rush, you don't you don't feel competency to continue providing non-pharmacologic managements which, at times, needs the patients to be thoroughly assessed and evaluated (GP4)*  *Usually, it’s tapped onto a consultation about something else. They’ll say, “Oh by the way, I also have pain in my knee,” and that’s just because of the type of people I see who are complex and got lots of other medical issues. (GP1)*  *Sometimes it’s a by-the-way diagnosis. They come in for five different things and then mention that sore knee if they do a bit of a history, and maybe examine and say, “Oh, that sounds like some osteoarthritis,” quick spiel, goodbye, and I’ve already done four other things (GP5)*  *So I go through with a 15-minute consultation, they come in with, “Oh, by the way, my hip is sore and I’ve got to have –” (GP14)* |
|  | Limited awareness of referral pathways and specific OA services | *I’ve never heard of it [GLA:D]. (GP23)*  *No, I haven't heard that one [GLA:D]. (GP20)*  *GLA:D? No, if you can enlighten me, what’s GLA:D program? (GP18)*  *I don’t know anything about those services [OAHKS and GLA:D], so – no (GP6)*  *Oh, no. What’s GLA:D? (GP7)*  *Nope [haven’t heard of GLA:D] (GP24)*  *I don’t know, group exercises programs or something. I don’t really know. And I don’t know what we have in our area. (GP23)*  *I’m not really aware of this service [GLA:D] (GP9)*  *Yes [have heard of GLA:D], but I don’t know much about it. (GP15)*  *No [Not heard of GLA:D]. What is that program? (GP13)*  *I heard of the term [GLA:D], but I don’t know what it is about. (GP11)*  *No [not aware of GLA:D] (GP4)*  *Yeah, I do know about it [GLA:D] . . . I know it’s a well-organised exercise program run by physiotherapists, group program, which has proven efficacy. We have patients from our clinic who go to it as well and some providers here in [Regional town A] and [regional town B] who do it. I think it’s, yes, its another way of providing education and exercise. (GP22)*  *Yeah, I have. [heard of GLA:D] (GP2)*  *No, what’s that? [GLA:D] (GP25)*  *Yeah [have heard of GLA:D]. My sister is a physio so, yeah. (GP12)*  *Yeah, I don’t know anything about it [GLA:D]. I’ve heard of it. So that’s probably one thing but, honestly, I can’t even remember what it stands for. Oops. (GP1)*  *I think I might have heard of it [GLA:D]. I might have heard of one of the physios. I’ve heard a bit about it but I don’t have the deep understanding of it, or it’s not something that I know the contents of the program but that is something, perhaps, is it for osteoarthritis? (GP16)*  *Yeah, I have done, I sent my own mum [to GLAD]. (GP5)*  *No [not heard of OAHKS] (GP9)*  *Heard about it, yes, but I have never used it. (GP20)*  *Do we have osteoarthritis clinic in every suburb then we can know that people do. Because I used to have a good physio in Perth. I found them because I’ve got a friend of mine who works in GP and orthopaedics . . . I'm from Perth, so now nearly two and half year in Melbourne. I don't know where to refer. (GP8)*  *So, good if that’s happening, but I’m not sure about [XX health network] catchment area. I’m not sure if that’s happening in our area. Sorry. (GP7)*  *Ah, no [not heard of OAHKS] (GP21)*  *I don’t know what the OAKHS clinic is or whether we have them. (GP23)*  *I've heard of OAHKS but it's not on my usual – when I tried to refer electronically, I don't see it pop up. So, no, I don't know too much about that. (GP24)*  *Mm-hmm no [not heard of OAHKS] (GP15)* |
|  | Enabler to referring for physiotherapy -knowledge of outcomes of programs including patient handouts | *So I think if you can give a specific example like, “I want you to see the physio for this specific thing,” that’s really useful. (GP3)*  *So I think I see that as probably the main barrier to non-operative treatment, is probably the patient seeing the benefit or seeing the utility in it, and then getting the motivation to go and do it. I think if they could see the utility in it and how useful it is, you could say to them, like, maybe if there were simple stats or something, you could say, “Look, this many percentage of people, if they do this program, this is what happens,” it’s having some information like that to help the motivation, to help them see the utility in it and then that, in turn, hopefully, will help the motivation that if you can see something will be useful, I think it can just feel a bit like you’re throwing all these things at them and they’re a bit like, “Will any of this help out?” For instance, to see the physio, feeling a bit like, “Well, am I just going to the physio for the sake of seeing physio or actually, it’s gonna be helpful?” (GP3)*  *I think … yeah, maybe having specific programs is really good. So I think the GLA:D program is great, ‘cause it’s something that is specific. And having – seeing physios who know the – I guess that’s the other thing, going to see a physio who knows what they’re doing and is knowledgeable, seeing a GP who is knowledgeable about things, because if you’re going to see someone and they just – you feel like they’re just throwing some random ideas at you, that’s not gonna be very – feel like much of a plan and you’ll probably a lot less likely to comply. (GP3)*  *Maybe if there’s a better way for them to understand like how physio is going to help, maybe some material we can give them, like proper case studies that we can discuss with them about – yeah. And the statistics that we can say like this many people have avoided joint replacements by doing this physio program. But I wouldn’t know what – I’d have to look up journal articles to know any of those statistics at the moment. (GP12)*  *Maybe some information. I don't know if there's any handouts that say, what you just said the GLA:D study or something like that but in a handout that is patient-friendly, something that they can easily read and go, “Oh, so if I do have surgery, this says that I'm probably gonna be equally happy if I don't have surgery and just do this physio program.” ‘Cause some people do think, incorrectly, that surgery is the answer and sometimes that you will set them back in terms of their quality of life. (GP24)*  *I think just being able to be persuasive and really selling it – have to be a salesman for it, really. I have to kind of really encourage them, persuade a patient like this is gonna make a big difference for them, and they have to buy into it. If they don’t buy into it, then even though you give them the referral, they may just not go. So, I think the more I – probably the more I understand about the benefit, the easier it is for me to be able to persuade someone to do something, the better I understand it – that’s with regards to any condition, really, as well as osteoarthritis. (GP6)* |
|  | GLA:D viewed positively | *So if we have a proper program in place to help with the patient’s management that they can benefit and the young people start to prevent arthritis is always critical for everybody's life. (GP9)*  *I’ve heard about that. Generally, most of the orthopaedics, they try to do that before the patient, just to try and avoid knee replacement. I don’t know much about it but it’s very, very popular. (GP21)*  *I think maybe I refer maybe around 10 to 15 patients and most of them are elderly, around 60, 70, and they have done really well with the program. I think it’s eight-to nine-week program, and people are actually – they are really happy with it but in three or four patients so far, those patients who are really going for knee replacement, and they could avoid it, so they are still doing really well with that program. Although they are not still doing it, but they’re all doing other exercises, non-weight-bearing exercises, and all that, which is working (GP21)*  *I know it’s a well-organised exercise program run by physiotherapists, group program, which has proven efficacy. . . It’s very worthwhile. (GP22)*  *Yeah, I’ve sent a few and they love it. I think their pain goes down and their function improves, and I think they really enjoy the one-on-one or the attention as well. It’s really nice. (GP5)* |
|  | Concept of advanced physiotherapy services mostly viewed positively | *it would be the best approach . . . where patients can access physio assessment and possible decision making as to how soon that the patient would benefit from seeing the orthopaedics. Anyway, with waitlist for the orthopaedics, they may not see the orthopaedics after a year or even sometimes they know don't how soon they will be seeing, so if they would be able to try physiotherapist, who would assess and classify them . . . whether the treatment can be given by the physio themselves or there is a need to see the orthopaedics as soon possible or how soon. That's a good thing to do because, as a GP, I just see the evidence for osteoarthritis diagnose, and try to manage on with both pharmacologic and non-pharmacologic approaches. And then it is always rush, you don't you don't feel competency to continue providing non-pharmacologic managements which, at times, needs the patients to be thoroughly assessed and evaluated but even I don't think, as a GP, we are trained in that manner. So I would say someone in between GP as a primary care and the hospital system who would facilitate and classify and treat accordingly or promptly refer if need be, is a good approach. (GP4)*  *Often when I sent a person for evaluation for a replacement or some nature like that, they are automatically going to OAHKS unless they're too bad, and so I do get letters from the OAHKS clinic . . . It works for some people. (GP19)*  *In the last couple of years, I was contacted by advanced – until then, I was not having a great idea but they called and they actually helped the patient a lot. So they are the liaising people between GP and orthopaedics. So if they want to try and speak to someone to expedite the procedure, then we generally speak to the liaison officer who then puts them to the advanced physio, and the advances physio does the assessment and they are basically like a messenger between the GP and the orthopaedics, and then they refer the patient whom they think that they need surgery . . .That works really good. Because when I do the referral, it has been seen by orthopaedic reg and, generally, then he puts it to the – I’m not sure how it goes in the hospital settings now, but the advanced physio actually triages much better because they’ve got a right idea . . . So it was a really important role they play in actually triaging effectively while seeing the patient. (GP21)*  *Look, if that’s happening, I’m so pleased because patients getting something. If they can’t see the surgeon, at least a physio department is there to help them, or to guide them, to give them some hope or a plan. (GP7)*  *It’s very good actually. And I think even if, for example, because I think there’s also a bit of a psychological impact, because sometimes what happens when I have to see the patients, while they are waiting to see the specialist, of course, because they think the specialist is going to fix everything and they will get benefit. Once they enter into that . . . where they have physiotherapist, they have got access to the pain specialist, they have everything there, it does help them because they know that they are doing something and it is within the hospital. And then eventually, if it doesn’t work, they know that they are going to have something done in the long run. As compared to the – and I think they take it a bit more seriously when they are seeing a physiotherapist in the hospital setting rather than the community-based. So they actually tend to think that “Okay, we definitely need to work” . . . they generally are happy in going there. (GP2)*  *Yeah, definitely [agree with AMP model of care]. I guess it put less of a strain on surgeon because I know myself, I'm probably guilty of referring to some of the patients who are clearly not surgical candidates, but whether because it's on patient assistance, or whether I just have no other options like they've tried everything and that's the last thing and they’re still complaining. But I think if there's a way where we can triage those referrals, so surgeons don't get flooded with every arthritis every knee, every hip, I think that would be good. (GP25)*  *So that is a very important role what they play, and then I think them to be taking charge of everything. So a couple of the patients, recently, one patient had hip replacement done which I thought she will never get it done, and that went through the advanced physio. She went there, she were assessed by the advanced physio. I’ve got a very detailed report which I’m really impressed with, and that patient went from triage category three to one. They have got a very important role in actual discrimination of the functional capability of the patient and also with the exactly what triage that they would need, so it would be much better. So they do play a very important role, definitely. (GP21)*  *Think that’s good if patient can be seen by the physiotherapy team in a timely – if they can be assessed, say, in couple of weeks after referral rather than – ‘cause often in the public system, I guess the waiting period could be couple months. If this doesn’t really change, doesn’t interfere the total waiting period – so I don’t want patients to be seen by the physio team after couple of months and then decide, “Okay, you don’t need to see orthopaedic surgeon.” Then the patient will feel like, “Okay, I have waited so long.” But if they could be triaged at very early stage, yes, that’s definitely gonna be helpful. (GP9)*  *Well, I think it's pretty good. So lots of situation, they probably need to have multidisciplinary team to inform, to assess, and lots of explanation, lots of reassurance, answer a lot of their queries, and then only until last state, they probably did see a specialist to do operation or anything like that. Yes. (GP20)*  *I think that would be better because not all issues can be solved with surgery. If we can avoid surgery and if you can be assessed and manage conservatively, that would be better. It avoids unnecessary costs in surgeries, and time, and pain, and all that. (GP15)*  *Of course, definitely [OAHKS is a good service]. Anything in the public system is good service to have. I mean, the public system is overwhelmed and we are the people who feel it. And up to the point that you just don’t know what to do and how to help these people, especially now. Yes, going back to the OAKHS clinic – yes, definitely. Any help is good help. (GP13)*  *Oh Yeah, that would be amazing. I mean I’ve got no ego issues. … I’ve got no ego issues about, if patients all went through a physio, got assessed, and then sent to whoever then gets to ask to prescribe or sending them off an imaging or an orthopaedic surgeon ‘cause they’re at that stage and get something done. So, I definitely see the value of that. (GP5)*  *So I have seen people who have gone and been assessed in that clinic and I think it's pretty appropriate actually. It’s a good way of- Because a lot of people will come to us – and some people won't even want to be physically-assessed when they want a referral to an orthopaedic surgeon . . . So it's probably an extra layer of intervention prior to needing surgery and I'm aware of the outcomes of hip and knee replacements, and that's that one-in-five are unhappy with the outcome. So it's pretty high rate and it's certainly not a perfect procedure and it's dangerous. So yeah, I think it's excellent. (GP10)* |
|  | Few GPs not in favour of AMP service | *Yeah, I think I've had a patient get knocked back through the public like that. I mean the whole point of me saying that, “I'm referring for surgery,” is because I feel they need to get a surgical opinion. So for me, I don't see the point of getting a second person to say yes or no. It's almost like doing it twice, my personal opinion (GP24)*  *It works for some people. I wouldn't say that it works for everyone. And again, I might be wrong in this, but the way I understood the purpose of OAHKS, at least, is postpone the need for surgery. That is all I found they actually achieved and they probably would have better data to suggest what difference it's made, but almost all my patients who were under OAHKS ended up getting their replacements done. The question before me is did it postpone the date for surgery. (GP19)*  *Good and bad. I actually think it’s fine but often the patients don’t – ‘cause I think, like, you guys can do a good functional assessment, but the patients always go to a hospital with the expectation that they’re gonna be seen by a doctor or a surgeon, so I get really negative feedback from the patients. Even though I’ve warned them that when you’re referred to an orthopaedic surgery for a joint replacement, or a joint problem, you’ll probably be reviewed by a physiotherapist first. They get in their mind that they’re going to see a surgeon and then they ask – they come back asking to be re-referred. (GP12)*  *Sometimes despair that I sent someone to a public hospital. They get sent to the physio. The physio sends them then makes an appointment straight with the ortho and I’m just thinking it’s just making something hard that isn’t. But I do understand that there are people who are referring too early, so I just suck it up. (GP17)* |
|  | GP views on a potential community-based advanced physiotherapy service | *Perfect, perfect, absolutely. (GP19)*  *That would be good actually. Definitely. Definitely. With the community physio, they generally don’t have any links with a major hospital. They just send back to us the report on what the things, blah, blah, blah, but that could be really good, if there’s a link like that. That will expedite a lot of cases and just the genuine ones for the surgical doctor to actually review. Yeah, that would be really good if we have those types of services in the community. (GP21)*  *That will be very good. Say, when I diagnose a patient with osteoarthritis, and if it is mild to moderate, and I can refer to the physio in this system, so that they will give them a plan. “Okay. So, we are doing this and that. But after these steps also if you don’t see any improvement – okay, we will forward you to a surgeon.” So, that kind of a hope – so, patients are very happy for that. That would be very nice. Yes. (GP7)*  *Yeah, I think it would be handy. (GP9)*  *I think it will be very good. Actually they don't need to go to the hospital. Even the community health setting is more convenient for the patient as well, so not everyone rush into the hospital, find a parking, and they always have a mobility, especially for the oldies, to have a mobility assist, parking, walking, and finding their way is always very difficult in a bigger complex. (GP20)*  *Well, that would make it more accessible to a lot of patients and avoid the unnecessary hospital attendances. Yep. (GP15)*  *I completely agree with this, absolutely. Because, as I said, the minute you mention going to the hospital, the people's perception about hospitals is feeling that they are already end stage or debilitated, people get confused more the minute you mention referring to the hospital system. It is very hard to get to know how long you’ll have to wait to hear from the hospital system . . . And hence, if there is an intermediate body who would, as I said, assess and clarify on the steps and stages and the care model and what will be done in the next year or two, in collaboration with specialists from the hospital or from wherever, that would that would be the best approach, especially in terms of the quality of the care, the hierarchy of the care, and the triaging system, as well as the access to the allied health, like physio, in the meantime, or until they see the surgeon or sometimes they may not need to see the surgeon in the area. (GP4)*  *Yeah, I would [be in favour of a community OAHKS]. If the physios, or whoever was doing this triaging and assessments, do that all the time, I think that's really appropriate, and if there's pretty regular communication with the surgeons as well, they'd have a very good feel for who should move on to see a surgeon. (GP10)* |
| Patient level | Patient misconceptions  /beliefs exercise not helpful, surgery only option | *And a lot of them think that a knee replacement or hip replacement is the best thing since sliced bread and will make everything go away, and I point out to them, “There is nothing as good as their own joint when it comes to function and so on.” And really what we’re talking about is pain management. So they can’t think a joint replacement is going to be purely fantastic and then they’ll have an absolutely normal knee back. It doesn’t quite work that way. I think some of them are a little bit surprised by that (GP17)*  *I think people don’t quite understand the value of physio and exercise. So partly ‘cause pain equals damage, so thinking that that’s people don’t wanna think about, doing anything that involves exercise which obviously physio does involve that. So I think that’s part of the misconception of that . . . I think it is a bit of a mindset thing for some people about just not wanting to maybe proactively do something. They just want to passively have someone fix them. And so challenging that mindset I find difficult, I think, and they probably find difficult as well. . . And then people wanting a quick fix as well just explaining that you can do a replacement but that’s not simple either. So there is no quick fix but there are certainly ways that we can help like it takes time. (GP23)*  *I think until there’s a little more understanding of what hip and knee OA is in the community, it will come overtime of course as people don’t always get surgery. That’s the main stage of trying to convince people. (GP14)*  *Because patients aren’t really understanding what physios are meant to do. They often think they’re getting a massage or something. And you’re like, “No, no, you need to do the exercises, you need to – they’re not there to massage you or that’s not main thing. They actually – it’s much broader than that.” (GP3)*  *It’s about how can we enable them to do that [exercise] in a safe way where they don’t feel like they’re gonna ‘damage’ their joints. (GP1)*  *Sometimes they have these pre-conceived notions that if you've got arthritis, that one day you'll have a replacement and that it's a surgical option, so having to educate and breakdown some of those barriers is part of the issue as well. (GP10)*  *I think, you know, misconception between what an actual injury is and what arthritis soreness is, stops a lot of people from exercising (GP12)*  *Not perceiving that the physio can do anything, not doing what physiotherapist will ask them to do; so, therefore, they perceive that physio is not doing anything. Yeah, thinking – wanting – a lot of patients want surgery and physio doesn’t cut it (GP12)*  *So that’s their expectation, they’re coming in thinking they’ve got that wanting me to confirm it with an x-ray and send them off for a surgical opinion. (GP14)*  *Even yesterday, a patient said “Look, you told me I have osteochondritis or runner's knee, but I'm not sure because I'm a sport guy, I need to see a specialist” (GP8)*  *And I guess some people just want to go straight to a surgeon to get a definitive thing (GP23)*  *A lot of the time, they say, “Oh, I've just got bad knees, there's nothing to do about it. More physical activity might make it worse,” (GP15)*  *In my experience, patients think that the expectation is, “I've got arthritis, I wanna be pain-free whether it's by medications, or whether by surgical intervention,” that's the expectation. Not many people think about with a physio or some non-operative management will be useful or beneficial ‘cause I don't think they know that is probably first or second line in terms of management of arthritis, so I try to educate them about that. But I think it's really changing the patient's mentality that arthritis, unfortunately, is a chronic thing. Unlikely they will get you pain-free, and it's really changing expectations about how we manage arthritis. (GP25)* |
|  | Percevied expectation of management  and referrals | *People will tell you, “I know what you should do. I just want hands-on. Okay? So, give me a sheet or give me – I don’t want to see a dietitian, I know what she’s going to tell me. Just give me something to be hands-on.” (GP18)*  *So many people in the community have their own osteo, or chiro, or physio they normally see, not all of which have the appropriate GLAD qualifications and accessibility to someone with those qualifications. (GP14)*  *Sometimes it’s challenging. They come up with, “Ah, look, I know an osteopath, can I go there?” Then I can’t say no actually. Look, I probably know a bit about or maybe less but at least something about what you guys do and how it can help the joints, but if you ask me what a chiropractor or an osteopath could do, I’m like – I don’t know. But still, when they come and ask, “Ah, look, I know someone – they can do some good to my joints. Can I go and see them?” So, still, I can’t say no. “Okay, all right. You can go and see. (GP7)*  *I think one thing is that they come to see your certain expectations, so that can be a barrier. The neighbour across the fence has just had their knee done and they see this person walking with a limp and so we should get a knee joint replacement. I’ve had a lady last week who, been seeing at our clinic for quite some time, who was walking with a limp hasn’t got too much pain, has had some locking, not too much, family is basically convinced her that she needs it done, and from our advice, she’s gonna proceed with that. So I think the pre-existing expectations make it hard . . . also if they don’t have a belief in exercise or the benefits of specific exercise, or physiotherapist, or they may have seen practitioners of slightly different ilks who are a bit more concentrate on hands-on things rather than prescription of exercises, that makes it hard. So those factors are certainly very significant barriers. (GP22)*  *I'm probably guilty of referring to some of the patients who are clearly not surgical candidates, but whether because it's on patient insistence, or whether I just have no other options like they've tried everything and that's the last thing and they’re still complaining. (GP25)*  *The patient expects if they come in and they’ve got hip or knee pain, that they’re going to be referred to a surgeon and most of them are fully expecting that they’re going to get a joint replacement. So that’s their expectation, they’re coming in thinking they’ve got that wanting me to confirm it with an x-ray and send them off for a surgical opinion (GP14)*  *But, again, people would have a set mind and they want one thing in their mind and they might ask for chiro and chiro is not good for the knees, or they might ask for masseur or myotherapy and they are not option for them. (GP18)*  *And I guess some people just want to go straight to a surgeon to get a definitive thing which is fine. If that’s really what they wanna do then I’m happy to facilitate that. But I always bring it up and then I think it mostly how the patient responds as to whether they then go down the physio pathway. (GP23)*  *People usually have their own view about which branch of the allied health service they had preference for (GP16)*  *They make decisions as to how much and what they want to do. . . . I work with very high health-literate people and they make their choices. I can point them in the direction, I can tell them maybe what needs to be done, but ultimately they make the choice. (GP17)* |
|  | Lack of motivation to exercise is a barrier, while being motivated to exercise is an enabler | *In long term health issues, which is common to these patients who might have complex comorbidities, so getting the motivation to go and do exercise is really hard. And then they don’t do it, it gets worse and then exercise becomes almost impossible because they are in so much pain. (GP3)*  *They might not have that required mindset to actually go for it, because it goes for maybe eight to nine weeks, they find it a little bit daunting, especially the elderly group… The only thing is the compliance could be an issue. I would say that would be the main barrier. (GP21)*  *So are they actually going to have time to engage in a program because it’s a waste of time for the people who are delivering the program as well, if the patient isn’t interested, and they just did it because the doctor told them to, or not doing it because the doctor told them as well. (GP1)*  *Because with the physiotherapist, you really don’t see benefits straightaway. It’s not a quick fix. It’s like over a period of time, and the patient has to show their commitment and do their part of the job. (GP2)*  *They have to make a choice in balance of their life and work then spending time for their health. (GP8)*  *The patient’s willingness to actually go and do anything, because a lot of the time, they say, “Oh, I've just got bad knees, there's nothing to do about it. More physical activity might make it worse,” so a lot of the time, it's the patient education and the willingness for them to actually go for the physiotherapy. (GP15)*  *So, I do tell them [about exercises] but then some of them are very good in following it, but some really want somebody else to do the job. (GP2)*  *But I haven’t really had anyone that’s appropriate for it or that’s willing to do it [GLA:D].*  *Because it’s – they don’t want to commit to the sessions. They want to do it in their own time and way and it’s not convenient. (GP12)*  *“Do they want to?” and there is a certain age group or type of patient that they just want a pill, they really don’t wanna do much more. So you’re just not gonna get them out anyway. They really don’t wanna do anything for themselves. (GP5)*  *I guess motivation – I’ve got one guy at the moment who’s got osteoarthritis with his knees and he’s a young builder and he’s got massive quads wasting, but he’s – I keep trying to get him to see the physio, but he just doesn’t want to go. So, I guess it’s something to think about, but some people just don’t feel they’ll benefit . . . I guess motivation is a key thing . . . One of the reasons ‘cause he has seen an osteo. I think he quite likes the hands-on stuff, but he’s just not interested in following a program or strengthening. (GP6)*  *I think it’s a lot of patient’s motivations is a big factor, especially with things like seeing the physio, doing the exercises that the physio gives you . . . So, I think that’s the most challenging thing is, you have to have motivated patients, and it’s hard if you’re – I think that’s the hard thing as they all feel – they’re all in pain, or not all, but often they’re in pain, and so when you’re in pain, your instinct is to not wanna do anything, but then the actual treatment, you’re better off with if you’re active, if you’re engage in physiotherapy and exercise or whatever it is. So, getting the motivation to do that is really hard (GP3)*  *So I think I see that as probably the main barrier to non-operative treatment, is probably the patient seeing the benefit or seeing the utility in it, and then getting the motivation to go and do it. (GP3)*  *Sometimes it's the patient themselves not necessarily wanting to do the work, and a strength and exercise program (GP10)*  *So the challenge is not mine, it’s actually more theirs [patients]. They make decisions as to how much and what they want to do. And that choice they make when they come back and say, “Well my knees are still sore,” you’d start talking about the exercise program, and then they say, “Well, I never got to the GLA:D program.” That’s the time then to say, “Well, maybe it’s time. This is causing you issues. Maybe let’s get a move on.” But I work with very high health-literate people and they make their choices. I can point them in the direction, I can tell them maybe what needs to be done, but ultimately they make the choice. (GP17)*  *Not perceiving that the physio can do anything, not doing what physiotherapist will ask them to do; so, therefore, they perceive that physio is not doing anything. Yeah, thinking – wanting – a lot of patients want surgery and physio doesn’t cut it. (GP12)*  *Also if they’re motivated, that will make it easier to do that [engage in exercise]. So if someone believes that they need surgery because their mother needed surgery, they’re unlikely to engage in that. (GP1)* |
| Environmental/social level |  | *Transport is an issue, availability is an issue, I would say that would be [some of] the main barriers. (GP21)*  *Patients don’t have access to physiotherapy as often as they would like, then again – and plus, it’s also going to some other place and having things done and then coming back, a bit of logistics problem, financial issues, access to the facilities and having enough of the sessions, so a bit of everything. (GP2)*  *Accessibility, where is it, whereabouts (GP4)*  *Access, availability, I guess that’s one and the same. I suppose here, there’s a limited number of dietitians, and so getting them to be able to have an appointment is one thing. (GP22)*  *So, if it comes to having access in the [community health] services, I would say, look, what – you need to go and see someone so that you can get some benefit and something that you can keep on doing, more like a continuity rather than just going one-off and thinking, “Okay, it’s just too far, I can’t really go there” and things like that. (GP2)*  *I think access, and a few things like transport, elderly patients getting actually to the physio sessions can be quite difficult if they don't drive or they've got other comorbidities. (GP25)*  *Most people, like we’re about 40Ks from [regional town]. There’s a very good physio in [small regional town] and there’s an osteopathic clinic in [small regional town] too but, otherwise, people will generally need to travel to [regional town]. So for people who are elderly, that can be a barrier, just getting there. (GP16)*  *I suppose location and how accessible those services are as well. Probably not as big of an issue in somewhere like [a large metropolitan city] but in more regional areas, that might be an issue. (GP10)*  *I find it's very hard because they can't move around, who is gonna carry around, especially for the oldies, there’s no one, even with the relatives, how are they gonna go around. That's another issue. . . But a lot of them, nowadays, they said, “I can't come. I just need a phone consultation today.” . . . They said, “I just can't get to the clinic.” There's always bit of social issue there. (GP9)* |
|  |  | *And sometimes patients have got their own factor, like they cannot go at the time when the physio is available, especially at the early ones (GP21)*  *Some of them may not be even able to drive because, in the area here, grandparents coming from overseas and not able to drive around or some of them even may not have Medicare as such, and hence quite a variety of – especially the population around the area I work, the older people with osteoarthritis and musculoskeletal conditions from overseas but living here with their children, and accessibility is one issue that I've noticed, they have to abide by the schedules of their kids (GP4)* |
| Policy/  system level | Cost/lack of affordable physiotherapy | *So I will discuss it [physio] with everyone and then whether they would, I think, decide to go ahead with that, often – depends on finances . . . I think it’s management in a way that is financially accessible. It’s probably the thing I struggle with the most. (GP23)*  *Most of the physio, or osteopaths, or the chiropractor, they charge a gap as well (GP20)*  *And cost as well. I guess a lot of patients with arthritis are the older population, retirees, pensioners, healthcare card holders, (GP25)*  *You know, it actually doesn’t cover much of the cost and then you’re gonna be out-of-pocket like $80 to $90, or however much a physio session. (GP3)*  *And they can’t always afford physio more than five sessions that Medicare allows for. (GP19)*  *So generally the barrier would be limited visits, first of all. You don’t get much through EPC . . . this is a problem in the last couple of months is bulkbilling. It’s a bit hard to find someone who bulkbill with the care plan but, fortunately, we do have a few options with that in which we don’t have any barriers like that. So we’ve got a physio which comes to the practice and she bulkbills. So that makes it very easy. (GP21)*  *I would try as much as I can to do a care plan, so that patients at least get some benefit, but* *I explain that there can be a gap and all these things as well . . . And care plan – five is not enough . . . So, another thing is, of course, the finances. Actually, we were a bulk-billing practice. Now, we moved to mixed billing recently. So, most of our patients, they’re not very wealthy. So, even when we say 60 dollars, 70 dollars a gap, they would think twice. “Do I really have to go or can I manage with some painkillers?” So, that’s another thing (GP7)*  *I think one of the biggest concern would be the cost. Even though with GPMP [subsidized allied health plan], will get five visits and the government rebate, but I think these days, you still have to pay a lot of gap fees. Although I don’t really have the prices, I don’t really know how much they charge, but I just – under the impression if the patient has some sort of financial burden, they will just stop going after a few sessions. (GP9)*  *And then second of all is the cost. A lot of them are either retirees or parents of international students, or working visa holders, so they don't have that much healthcare overall, like security, in that sense (GP15)*  *So it is very common that people even asked about costs the minute you mentioned about referrals and continuity of care with physiotherapy. (GP4)*  *I think cost factors, some of them may have to pay some out-of-pocket costs and patients are sort of used to getting everything for free now that they don’t want to pay or they try not want to, so they rather avoid going or they try to use something at home, they try to manage themselves. (GP11*)  *It’s really hard because I think a lot of it comes down to time and money (GP12)*  *Cost is part of it as well. So sometimes we send people off to physios and it’s a fairly big out-of-pocket cost too, so they want something that they can do at home or without a cost. (GP10)*  *And of those who do, sometimes it really is just expensive . . . especially if they’ve got the extras, usually they consider – if they can afford insurance, they can afford to see a physio or dietician, or whatever. (GP5)*  *One thing I didn’t really touch on was the cost barrier. You know, not everyone can pay to see a physio or going to hydrotherapy, or whatever it is. (GP3)*  *It might be finances as well I guess in terms of being able to afford to go for physio. (GP6)*  *So basically, Medicare, only GP management plan and team care arrangement only allowed them to do five times. (GP20)*  *So having access to five sessions, when you think about it, it’s not many, especially if you’re trying to use that across multiple allied health sessions and you’ve got diabetes or you’ve got some other issue and you need to see a dietitian. (GP3)*  *And the EPC, I mean that’s only five visits per year. (GP5)*  *Those five visits of EPC [care plan], you got huge chunk to share it. There is almost a big crowd to share it. You got your podiatrist. You got your physio, then your dietetics, maybe one session or one or two. Often they’ll say, “Okay, I know what to cook or what to eat.” So, one is there. So you got one for the dietician, and two or four podiatrist. Then what are you left with? Three. So what are you gonna do with the physio? So, it’s also not enough. (GP13)*  *The other options are sort of using the EPC plan and they only get the five sessions with EPC in 12 months, so there’s not a lot of sessions they have to more less manage with doing the exercise in between, unless you want to pay for more sessions so that’s the other thing. (GP11)*  *The next problem comes when we’re referring them for the allied health services, and because there are only five visits in the whole calendar year, that’s the trickiest part of it. (GP2)*  *It’s really good that they can get access to five sessions through a care plan but sometimes it's actually they need more sessions and more so ongoing sessions with physios or allied health, that might benefit them long-term, but I guess five sessions over a calendar year is probably not enough. And some of them have said they've been good but after I stopped seeing a physio are regressed. (GP25)* |
|  | Waiting times for subsidized community allied health | *Well getting appointments, especially to the [community health services], sometimes they might have to wait for the community physio. (GP11)*  *Community health is really hard to get in with, so I use community health when we have to and we sort of just have to wait for the service to be available. (GP12)*  *Significant. Significant [barriers to community health referral]. I think the biggest barrier is delay (GP19)*  *I guess that can sometimes be a little bit difficult, is finding someone that the patient feels that they can see that’s local that I can access quickly (GP14)*  *If I needed to, I know there’s a [community allied health service] that I would refer to. And if there’s the option to, I would. Depending on the wait times and things that could also be a bit of factor. (GP3)* |
|  | Waiting times for public orthopaedic clinics | *I think even getting into OAHKS is such a nightmare these days. I mean the waitlist to be even considered for those things is ridiculous. (GP19)*  *if this is a patient who does not have private health cover, they can’t afford the private fee then, yes, we don’t have any other option than going for public. But I do tell them that it’s going to be a very long waiting time, so that they are aware of it. (GP2)*  *If they don’t have private insurance, then I will send them to public system. Unfortunately, public system waiting time is very, very, very long. So, often, it’s not only one referral. So sometimes the patient will come and – “I’m still waiting.” and you have to rewrite again, and quite often, it’s a lot of, we are under a lot of pressure, the patient who’s coming – okay, they call, they will tell you – I call the outpatient clinic. There was a nurse – nurse telling me that there’s only one way you can make this urgent – “Ask your doctor to write that it’s urgent.” So they come and they will ask you to do these things. Then you’re feeling embarrassed. You can’t write urgent. This is not a life-threatening condition. So you’re trying to find a way to explain this and it’s a long ongoing battle. (GP13)*  *I think it's really wait times. That's the biggest barrier for me, because currently for example, in [local health service], average consideration time for a hip replacement is 18 months. And a lot of them, because they're already advanced, they don't even get sent to OAHKS clinics, so they just sit there waiting, waiting, waiting (GP19)*  *And then I refer to a couple of hospitals, that’s the another thing, . . . So wherever the patient gets into first so that sort of thing. (GP21)*  *But, generally, we start a lot early. So what I do is if I know a patient who got bicompartment involved so like medial and, or some sort of progressive arthritis, I do those referrals because I know the waiting times can be long. . . . the only thing to be careful about is that you start it early, . . . because the public system can take up to one year. (GP21)*  *But I can’t think of any recent experience that I got a good feedback from the public system saying that, “Oh, your patient is given a date.” So, no, they always say, “Your patient is waitlisted.” So, waitlisted means we never know when they’re going to get an appointment. So, yeah, we do, because sometimes patients ask about a specialist fee and when I say, “Oh, it’s going to be hundreds, only 70 dollars would be back into your account.” They ask, “Can you do a public referral?” So, I would do, saying that no guarantee when you are going to get an appointment. . . but regarding osteoarthritis, no, not in public. I have done referrals, but no positives. (GP7)*  *When I was in the hospital system, when I was working in ED, we’d see a fair few people who didn’t have private health insurance and certainly couldn’t pay for much at all and who’d be on waitlists for two years for knee and hip replacements and so they come into emergency. So I guess that’s probably my major experience, is just seeing people coming to emergency with exacerbations of pains ‘cause they’ve been waiting for two years on the waitlist. So I don’t know if that’s still true or if that’s an unfair assessment but that was certainly what I always seemed to see. (GP23)*  *Oh, my goodness, long waiting. Anywhere here a huge pile or list of waiting, and you can see that patients are desperate about the access to specialists and how long they wait. That's one of the major concerns that they raise. (GP4)*  *It’s crazy wait time in the public system… He would’ve been on the wait list for the whole time I worked at the practice. So, probably, at least a year or two, and even that seemed like that was reasonably quick. I was surprised he got seen so quickly through the public system, ‘cause I was expecting it to be many, many – you know, I know for some people it might be years and years. Whereas, he, I think, got seen – it was probably under a year then. Yeah, that’s a long wait. (GP3)*  *I haven't done many referrals through the public system, I know what the waitlist is like. (GP10)*  *Everyone is aware that to get an appointment in the orthopaedic surgery is a year to two years, and then replacement afterwards– with a few exceptions (GP14)* |
|  | Enabler-patients having private health insurance | *So, if somebody has a private health cover and they can afford it, I would definitely refer them for private (GP2)*  *So basically, there’s no issue [with waiting times for orthopaedics] with the people with private health . . . So people who have got private insurance, they don’t have an issue (GP21)*  *So, it’s mostly the private ones, they do get appointments – surgeries are done. So, things are happening in the private sector. (GP7)*  *So if there's no other option then they will go on a public waitlist, but most of my patients have private insurance. (GP10)*  *My demographic is usually the vast majority are in a position where they would go privately because everyone is aware that to get an appointment in the orthopaedic surgery is a year to two years, and then replacement afterwards (GP14)* |
