# Supplementary material for: General practitioners experience multi-level barriers to implementing recommended care for hip and knee osteoarthritis: a qualitative study
Source: BMC Prim Care. 2024 Dec 19;25:423. doi: 10.1186/s12875-024-02658-0 (PMC11657540; doi:10.1186/s12875-024-02658-0)
Supplement: Supplementary file 3 — Supplementary Material 3 [file 12875_2024_2658_MOESM3_ESM.docx]

Additional File 3: COREQ Checklist

| Domain 1 Research team and reflexivity |  | Info | Where |
| --- | --- | --- | --- |
| Personal characteristics  1. Interviewer or facilitator | Which author/s conducted interview/focus group | Interviewer and background outlined in data collection | Lines 89-91 |
| 2. Credentials | What were the researchers’ credentials e.g. PhD, MD | Credentials and background experience outlined in data analysis | Lines 89-91, 152-158 |
| 3. Occupation | What was their occupation at the time of the study | Physiotherapist, GP and academics Listed in data collection and data analysis | Lines 89-91, 152-158 |
| 4. Gender | Was the researcher male or female | Female, stated in data collection | Line 89 |
| 5. Experience and training | What experience or training did the researcher have | PhD candidate, 2 day qualitative research course and undertaken previous qualitative research, remainder of team experienced researchers. Listed in data collection and data analysis | Lines 89-91, 152-58 |
| Relationship with participants 6. Relationship established | Was a relationship established prior to study commencement | No previous relationship to interviewer outlined in data collection. | Line 91 |
| 7. Participant knowledge of the interviewer | What did the participants know about the researcher? E.g. personal goals, reasons for doing the research | Knew interviewer was physiotherapist and PhD candidate, knew was part of larger study (REFER-being published separately) looking at developing intervention to assist GPs referring to physiotherapy as per PICF and introductory email | Lines 455-457 |
| 8. Interviewer characteristics | What characteristics were reported about the interviewer/facilitator e.g. bias, assumptions, reasons and interest in the research topic | Physiotherapist, woman, working in osteoarthritis hip and knee service, undertaking PhD-Outlined in data collection and data analysis, limitations | Lines 89-91, 154-158, 455-457 |
| Domain 2 study design |  |  |  |
| Theoretical framework  9. Methodological orientation and theory | What methodological orientation was stated to underpin the study? | Philosophical orientation underpinning the qualitative approach was naturalistic enquiry, used an interpretive description approach.  Inductive thematic analysis was used for analysing data. | Lines 59-66, 134-136 |
| Participant selection  10.Sampling | How were participants selected? E.g. purposive, convenience, consecutive, snowball | Open to all eligible participants with snowball approach-outlined in participants | Lines 75-82 |
| 11. Method of approach | How were participants approached? E.g. face to face, email, telephone | Mix of email, telephone, advertisement for online expression of interest | Lines 76-82 |
| 12. Sample size | How many participants were in the study | Final Sample size 26 interviews, 25 transcripts included (one declined interview audio recording) recruitment continued until no further ideas identified, additional 2 interviews conducted to confirm this | Lines 82-84, 166-169Table 1 (lines 174-180). |
| 13. Non-participation | How many people refused to participate or dropped out? Reasons? | See table 1 for details of GPs/GP practices contacted, responded, declined, consented and attended interview | Line 166-169, Table 1 lines 174-180). |
| Setting  14. Setting of data collection | Where was data collected? E.g. home, clinic, workplace | Remotely via zoom or telephone -outlined in data collection | Lines 91-92 |
| 15. Presence of non-participants | Was anyone else present besides the participants and researchers | No one else was present during interviews, all interviews were 1:1 |  |
| 16. Description of sample | What are the important characteristics of the sample e.g. demographics, date | Demographics reported in text and table 2 | Lines 84-86, 189-172, Table 2 (line 182) |
| Data collection  17. Interview guide | Were questions, prompts, guides provided by the authors? Was it pilot tested? | Interview guide development described in text and included as Supplementary file | Lines 113-124, Additional file 1 |
| 18. Repeat interviews | Were repeated interviews carried out? If yes how many | No |  |
| 19. Audio/visual recording | Did the research use audio or visual recording to collect the data | Audio recorded-outlined in data collection | Line 91-92 |
| 20. Field notes | Were field notes made during and /or after the interview or focus groups | Yes |  |
| 21. Duration | What was the duration of the interviews or focus group | 15-52 minutes, average 29 minutes | Line 171-172 |
| 22. Data saturation | Was data saturation discussed? | Yes-in data collection | Lines 82-84, 125-127 |
| 23. Transcripts returned | Were transcripts returned to participants for comment/and or correction? | No -due to time constraints of interviewees-data analysis and discussion | Lines 132-133, 457 |
| Domain 3 analysis and findings |  |  |  |
| Data analysis  24. Number of data coders | How many data coders coded the data | At least two for each interview, a third read a random sample of 12 transcripts | Lines 130-132, 137-143 |
| 25. Description of the coding tree | Did authors provide a description of the coding tree | N/A |  |
| 26. Derivation of themes | Were themes identified in advance or derived from the data | Themes were derived from the data | Lines 134-144 |
| 27. Software | What software, if applicable, was used to manage the data | NVivo was used by one of the coders | Lines 139 |
| 28. Participant checking | Did participants provide feedback on the findings | No, although one member of research team providing feed back was a GP | Lines 153, 161-162 |
| 29 Quotations presented | Were participants quotations presented to illustrate the themes/findings? Was each quotation identified e.g. participant number? | Yes -examples included throughout results, and supp file table 1 and 2 with further examples | See results section, (lines 197-198, 201-205, 213-217, 223-226, 235-236, 239-241, 256-264, 271-279, 287-296, 299-300, 309-316, 320-322, 329-331, 341-344, 353-362; additional quotes in Additional file 2, Supp tables1 and 2 |
| 30. Data and findings consistent | Was there consistency between the presented data and the findings | Yes-see results with exemplar quotes, supplementary files for further example quotes | Lines 184-362 Figure 1, Additional file 2, Tables 1 and 2 additional quotes |
| 31. Clarity of major themes | Were major themes clearly presented in the findings | Yes-see results for two themes, second theme mapped to socioecological model (Figure 1), with exemplar quotes | Lines 184-186, 188-192, 243-248and Figure 1 |
| 32. Clarity of minor themes | Is there description of diverse cases or discussion of minor themes | Yes- see results section for discussion of minor themes. Examples of diverse cases included | Lines 184-362 for results including discussion of minor themes; Figure 1; diverse cases see lines 206-217, 297-300 |
